# Phage Paride can kill dormant, antibiotic-tolerant cells of *Pseudomonas aeruginosa* by direct lytic replication

**DOI:** 10.1038/s41467-023-44157-3

**Published:** 2024-01-02

**Authors:** Enea Maffei, Anne-Kathrin Woischnig, Marco R. Burkolter, Yannik Heyer, Dorentina Humolli, Nicole Thürkauf, Thomas Bock, Alexander Schmidt, Pablo Manfredi, Adrian Egli, Nina Khanna, Urs Jenal, Alexander Harms

**Affiliations:** 1 Biozentrum, University of Basel, Basel, Switzerland; 2 Institute of Food, Nutrition and Health, D-HEST, ETH Zurich, Zurich, Switzerland; 3 Laboratory of Infection Biology, Department of Biomedicine, University and University Hospital Basel, Basel, Switzerland; 4 Division of Clinical Bacteriology and Mycology, University Hospital Basel, Basel, Switzerland; 5 Applied Microbiology Research, Department of Biomedicine, University of Basel, Basel, Switzerland; 6 Division of Infectious Diseases and Hospital Epidemiology, University and University Hospital of Basel, Basel, Switzerland

**Keywords:** Cellular microbiology, Bacteriophages, Virus-host interactions, Pathogens

## Abstract

Bacteriophages are ubiquitous viral predators that have primarily been studied using fast-growing laboratory cultures of their bacterial hosts. However, microbial life in nature is mostly in a slow- or non-growing, dormant state. Here, we show that diverse phages can infect deep-dormant bacteria and suspend their replication until the host resuscitates (“hibernation”). However, a newly isolated *Pseudomonas aeruginosa* phage, named Paride, can directly replicate and induce the lysis of deep-dormant hosts. While non-growing bacteria are notoriously tolerant to antibiotic drugs, the combination with Paride enables the carbapenem meropenem to eradicate deep-dormant cultures in vitro and to reduce a resilient bacterial infection of a tissue cage implant in mice. Our work might inspire new treatments for persistent bacterial infections and, more broadly, highlights two viral strategies to infect dormant bacteria (hibernation and direct replication) that will guide future studies on phage-host interactions.

## Abstract (web summary)

Bacteriophages have primarily been studied using fast-growing laboratory cultures of their bacterial hosts. Here, Maffei et al. show that diverse phages can infect deep-dormant bacteria and suspend their replication until the host resuscitates; however, a particular phage can directly replicate and induce the lysis of deep-dormant hosts.

## Introduction

Unlike the rapidly dividing cells that may come to mind at first when thinking of microbes, most bacteria on our planet are in a slow- or non-growing, dormant state characterized by a low-energy physiology and high resilience to external perturbations^1^. This includes completely inactive spores—described as “the purest form of microbial dormancy”—but also a wide variety of quiescent yet vigilant states of low activity that are poised to resuscitation when nutrients or signaling molecules are supplied^2–4^. These dormant bacteria are usually seen as a microbial bet-hedging strategy to ensure population survival via the persistence of heterogeneous, highly resilient cells through unpredictable catastrophic events^5^. In many cases, bacterial dormancy is induced through a well-ordered physiological program in response to stress or starvation that also controls the “stationary phase” of laboratory cultures after exhausting the growth potential of their culture conditions^1,6^. For the model organisms *Escherichia coli* and *Pseudomonas aeruginosa*, these processes are largely orchestrated by signaling through the stress and starvation sigma factor RpoS as well as the second messenger (p)ppGpp^1,6–9^.

The antibiotic drugs administered in clinics constitute just another unpredictable existential threat that bacteria can evade through dormancy. While antibiotic resistance denotes the ability of bacteria to grow in presence of an antibiotic, the antibiotic tolerance of dormant cells causes a slower killing compared to growing cells because the cellular processes commonly poisoned by bactericidal antimicrobials are tuned down or inactive^10–12^. Therefore, dormant antibiotic-tolerant cells sometimes known as “persisters” can survive drug treatment and have been implicated in the resilience of chronic or relapsing infections^13^. Despite decades of intensive research, common underlying principles of these heterogeneous persister cells are still hotly debated and no effective treatments are available in clinics^10–12^. While new antimicrobials from classical in vitro research might help us fight antibiotic-resistant infections, they are likely to be as ineffective against antibiotic persistence in vivo as the regular antibiotic drugs that are currently available.

One promising alternative strategy to combat antibiotic resistance is the therapeutic application of bacteriophages (or short “phages”), the viruses that prey on bacteria^14,15^. Despite its long history, phage therapy has remained a niche approach in most countries due to technical difficulties and a notorious lack of reliability in clinical trials^14,15^. Already almost hundred years ago a dedicated study concluded that “the bacteriophage, which acts so well in vitro, does not have a similar action in vivo^16^”. The physiology of bacteria at the infection site is therefore a key parameter for phage infectivity and, consequently, for successful phage therapy, but the underlying molecular mechanisms are only poorly understood^17–20^. Analogous to antibiotic persistence, it is intuitive that the dormancy of stressed and starved bacteria in vivo might impair phage therapy. Previous work indeed showed that the productivity of phage infections is positively correlated with host growth rate and that fully growth-arrested cells are refractory to phage replication^21–25^.

Consequently, commonly studied virulent phages either avoid adsorption to dormant bacteria^26^ or hibernate in the low-energy physiology of these cells until nutrients become available again and lytic replication resumes^25,27–29^. The latter phenomenon is known as pseudolysogeny^30^ analogous to the lysogeny of temperate phages which can integrate their genome into the host’s genome, e.g., when they encounter starved host cells^31^. Nevertheless, we reasoned that phages with the ability to directly replicate on dormant hosts likely exist in nature given the abundance and diversity of dormant bacteria and the density of phage-host interactions^1,32^. Previous work indeed described a few examples of phages with this ability^33–35^ and reported cases of successful phage therapy targeting chronic bacterial infections^20,36,37^. However, the underlying molecular mechanisms and possible phage replication on truly deep-dormant, antibiotic-tolerant bacteria had remained elusive. Studying such phages would give important insights into viral ecology in nature and might open new avenues to treat chronic infections, e.g., by inspiring new treatment strategies to overcome the resilience of dormant bacteria.

In this study, we therefore performed large-scale bacteriophage isolation experiments to isolate new phages with the ability to directly kill antibiotic-tolerant, dormant cells of *Escherichia coli* or *Pseudomonas aeruginosa* by lytic replication. While most phages seemed to merely hibernate in these hosts, we isolated a new *P. aeruginosa* phage named Paride that uniquely replicates on deep stationary-phase cultures of laboratory and clinical strains of this organism. Intriguingly, we found that Paride can even sterilize deep-stationary phase cultures of *P. aeruginosa* if combined with the β-lactam meropenem via a phage-antibiotic synergy that also strongly reduces bacterial loads in a murine tissue cage infection model. Unexpectedly, the replication of Paride on dormant hosts largely depended on the bacterial starvation and stress response signaling that is also required for the antibiotic tolerance of these bacteria. This suggests that Paride specifically exploits weak spots in the resilient physiology of dormant bacteria that could be targeted as Achilles’ heels by new treatment options.

## Results

### Commonly studied bacteriophages can’t replicate on antibiotic-tolerant, deep-dormant bacteria

**Fig. 1:**
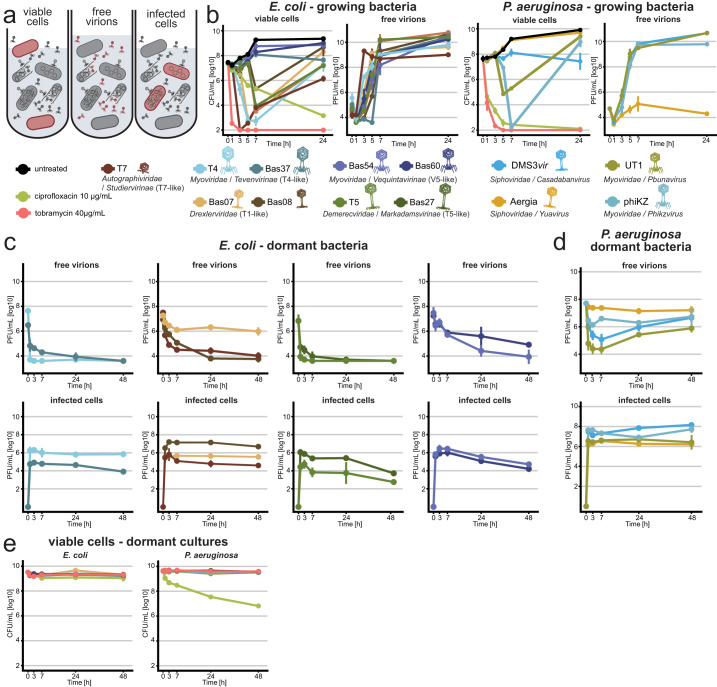
Most bacteriophages cannot replicate on deep-dormant *E. coli* or *P. aeruginosa*. **a** Schematic of metrics that were recorded during phage infection experiments. **b** Fast-growing cultures of *E. coli* or PAO1 *Δpel Δpsl* were treated with antibiotics or phages (MOI ≈ 0.001) and viable colony forming units (CFU/ml) as well as plaque-forming units of free phages (PFU/ml) were recorded over time. **c**–**e** *E. coli* K-12 MG1655 or *P. aeruginosa* PAO1 *Δpel Δpsl* subcultured for 48 h were treated with antibiotics or phages (MOI ≈ 0.01) and viable CFU/ml as well as PFU/ml of free phages and infected cells were recorded over time. Data points and error bars in (**b**–**e**) show the average of 2–3 biological replicates and standard error of the mean as specified for each dataset in the Source data file. Limits of detection are 2 log10 CFU/mL for viable cells, 3.6 log10 PFU/mL for free phages and 2.6 log10 PFU/mL for infected cells. Additional comments regarding the apparent rise of PFU/ml for some *P. aeruginosa* phages are included in Supplementary Note 1. Source data are provided as a Source data file.

We initiated our study by exploring the ability of multiple different phages including commonly used laboratory models to kill deep-dormant cultures of *Escherichia coli* or *P. aeruginosa* by direct replication. Given that well-chosen and strictly controlled assay conditions are crucial for meaningful experiments with dormant bacteria^10,12,38,39^, we had previously established a rigorous methodology that is based on a fully defined culture medium and enables work with both growing or non-growing, stationary phase bacteria^40^. In the current study, we have now performed whole-proteome analyses of these cultures during rapid growth and at different time points in stationary phase to further characterize our experimental system (see “Methods”). Briefly, our results confirmed the intuitive notion that the bacterial physiology shifts massively when growth stalls upon entry into stationary phase ca. 8 h after subculturing^40^ and then continues to change from this state of early dormancy while the bacteria become more starved and stressed until deep dormancy 48 h after subculturing (Fig. S1). To study antibiotic tolerance or phage sensitivity, bacterial cultures were then challenged with drugs and/or viruses during exponential growth or in a deep-dormant state (48 h after subculturing/ca. 40 h after entering stationary phase) and bacterial viability as well as viral infections were tracked over time^40^ (Fig. 1a).

In this setup, fast-growing cultures of *E. coli* and *P. aeruginosa* are readily cleared by antibiotic treatment and highly permissive to replication by all tested bacteriophages (Fig. 1b). Conversely, the deep-dormant cultures displayed massive antibiotic tolerance and did not allow replication of any tested bacteriophage (Fig. 1c–e). Instead, most phages rapidly adsorbed and then seemed to enter a state of hibernation in dormant hosts that is apparent as a stable number of infected cells over time as observed already previously, e.g., for *E. coli* phage T4 and *P. aeruginosa* phage UT1^28,29^.

**Fig. 2:**
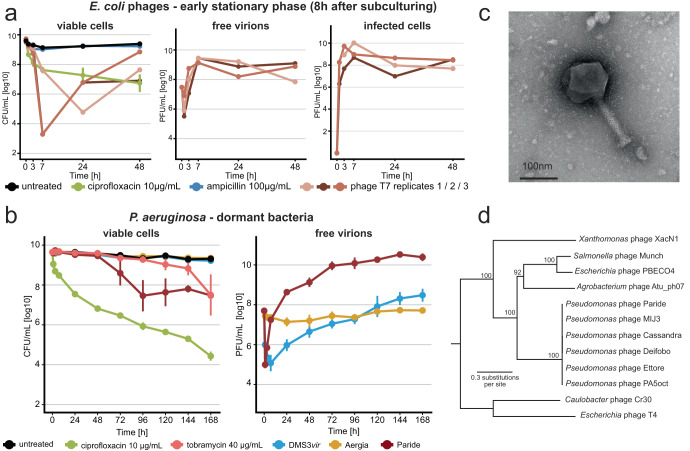
Paride is a new *P. aeruginosa* phage that can replicate on deep-dormant host cells. **a** *E. coli* K-12 MG1655 subcultured for 8 h were treated with either antibiotics or phage T7 (MOI ≈ 0.01) and viable colony forming units (CFU/ml) as well as plaque-forming units (PFU/ml) of free phages and infected cells were recorded over time. **b** *P. aeruginosa* PAO1 *Δpel Δpsl* subcultured for 48 h were treated with antibiotics or phages (MOI ≈ 0.01) and viable CFU/ml as well as free phages were recorded over time (see also Supplementary Note 1). All data points and error bars show the average of three biological replicates and standard error of the mean except in (**a**), where qualitatively similar but temporally shifted results of T7 infection experiments are shown individually. Limits of detection are 2 log10 CFU/mL for viable cells and 3.6 log10 PFU/mL for free phages. **c** Negative stain TEM micrograph of phage Paride. **d** Maximum-likelihood phylogeny of Paride and other group 2.2 jumbo phages as defined by Iyer et al.^42^ with phages T4 and Cr30 as outgroup (see “Methods”). Bootstrap support is shown if >70. Source data are provided as a Source data file.

Previous studies had highlighted the ability of *E. coli* phage T7 to replicate on starved, stressed, and stationary phase hosts^33,41^, but we merely observed hibernation of this phage when infecting deep-dormant cultures (Fig. 1c). Given the exceptionally long cultivation of bacteria in our setup before phage or antibiotic challenge, we suspected that the host cells in previous work might have been in a less dormant and, consequently, more permissive physiological state. To test this hypothesis, we generated analogous data using early stationary phase cultures treated either 8 h after subculturing (when cultures have just reached maximal density^40^) or 4 h later. As expected, the bacteria at these time points displayed an intermediate antibiotic tolerance that was higher than for growing cells but lower than our deep-dormant cultures treated 48 h after subculturing (Fig. 2a as well as S2a–c and S3a, b). Intriguingly, phage T7 stood out from all other tested *E. coli* and *P. aeruginosa* phages for its ability to replicate on the cultures treated 8 h after subculturing while no phage could replicate on the bacteria challenged 12 h after subculturing (Fig. 2a as well as S2a–c and S3a, b). These results confirm a special ability of phage T7 to replicate on some stressed and starved cells that exhibit intermediate antibiotic tolerance but clearly showed that highly drug-tolerant, deep-dormant cells were off limits for all previously tested phages.

### Bacteriophage Paride can kill deep-dormant *P. aeruginosa* by direct lytic replication

To isolate new phages that could replicate on these cells, we therefore resorted to the systematic screening of environmental samples using deep-dormant cultures of *E. coli* or *P. aeruginosa* as bait (see “Methods”). These experiments resulted in the isolation of bacteriophage Paride, a *P. aeruginosa* phage that rapidly adsorbs to deep-dormant host cells and then massively replicates, killing >99% of the bacterial population and causing the culture to lyse (Figs. 2b and S3c). Interestingly, Paride also proficiently replicates on growing host cells (Fig. S3d). The phage forms virions of myovirus morphotype and has a large genome of 287,267 bp, i.e., far beyond the 200 kb threshold defining “jumbo phages” (NCBI GenBank accession OR805295; Fig. 2c)^42^. Phylogenetic analyses revealed that Paride is a close relative of previously described phages PA5oct and MIJ3 (Fig. 2d)^43,44^. Conversely, Paride is not related to well-studied *P. aeruginosa* jumbo phage phiKZ which famously forms a “phage nucleus” in infected cells^45^ but cannot replicate on dormant hosts (Figs. 1c and S3a, b), and belongs to an entirely different clade of large microbial viruses^42^.

**Fig. 3:**
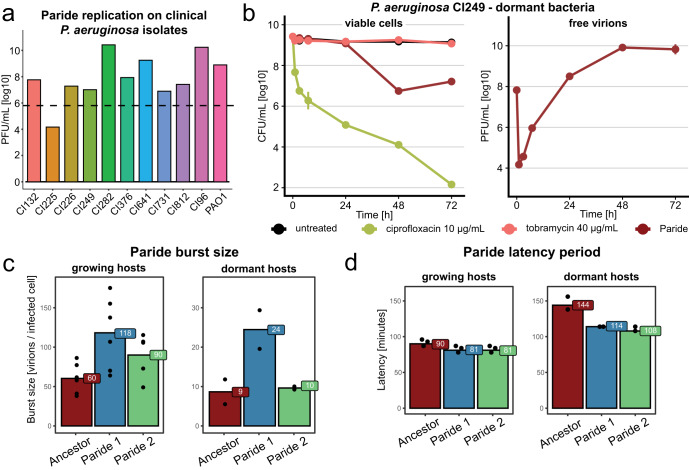
Infection of clinical *P. aeruginosa* isolates by Paride and one-step growth curves. **a** Free phage titers of Paride after infecting deep-dormant cultures of different clinical isolates of *P. aeruginosa* for 48 h (dashed line: inoculum). **b** *P. aeruginosa* clinical isolate CI249 subcultured for 48 h was treated with antibiotics or Paride (MOI ≈ 0.01) and viable CFU/ml as well as free phages were recorded over time. Due to lack of robust growth in M9Glc this experiment was performed in M9Rich (see Fig. S4a for a control experiment with the *P. aeruginosa* PAO1 *Δpel Δpsl* in this medium). Data points and error bars show average and standard error of the mean of three independent experiments. Limits of detection are 2 log10 CFU/mL for viable cells, 3.6 log10 PFU/mL for free phages. **c**, **d** Burst size and latency of ancestral Paride as well as two evolved clones passaged on deep-dormant cultures were determined by one-step growth experiments (see also Fig. S4b, c). Data bars represent the average of two (non-growing hosts) or six (growing hosts) independent experiments and all individual data points are shown. Source data are provided as a Source data file.

Repeated attempts at isolating different phages that can replicate on deep-dormant, antibiotic-tolerant bacteria exclusively uncovered diverse close relatives of Paride that we called Cassandra, Deifobo, and Ettore (Fig. 2d and Table S1) but no other phage, suggesting that this ability is very rare. We therefore explored whether the observed replication of Paride on deep-dormant cultures might be a laboratory artifact from the combination of this phage and the *P. aeruginosa* PAO1 model strain. However, Paride also readily replicated on stationary-phase cultures of different susceptible *P. aeruginosa* strains from a collection of clinical isolates (Figs. 3a, b and S4a), demonstrating that this phenomenon is not restricted to the PAO1 laboratory strain.

### Quantitative assessment of Paride infections and experimental evolution

We then performed one-step growth experiments to quantify the speed and productivity of Paride infections. For regularly growing hosts under our experimental conditions, we determined a burst size of around 60 (i.e., virions produced per infected cell, Fig. 3c) and a latency period of 1.5 h (i.e., infection time needed to generate new virions; Fig. 3d). When infecting deep-dormant hosts, Paride showed a reduced burst size of ca. 9 and a prolonged latency period of ca. 2.5 h (Fig. 3c, d). With view to possible medical relevance of Paride’s ability to replicate on dormant hosts, we sought to improve this ability by serially passaging two independent lines on deep-stationary phase cultures for around 600 generations (see “Methods” and Fig. S5). Both evolved lines improved burst size and latency, though the improvement of burst size was more pronounced in one lineage while the improvement of latency was more pronounced in the other (Fig. 3c, d). These results suggest that the two lineages improved overall infection efficiency by convergent evolution via different routes, though none of these improvements was specific to infecting dormant cultures.

### Paride targets the outer core of *P. aeruginosa* LPS as essential host receptor

**Fig. 4:**
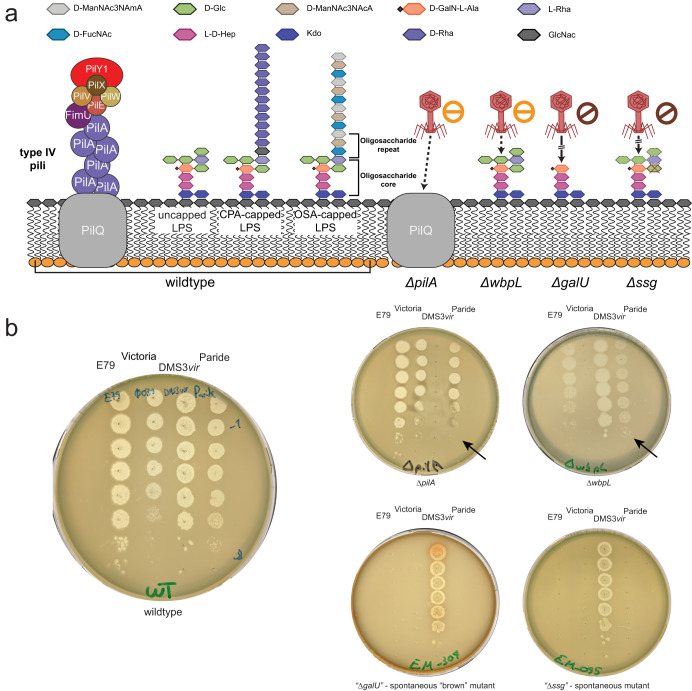
Paride binds the outer LPS core of *P. aeruginosa* as essential host receptor. **a** Schematic representation of the *P. aeruginosa* PAO1 *Δpel Δpsl* cell surface and the susceptibility of different mutants to Paride in top agar assays (see “Methods” for details). CPA = common polysaccharide antigen, OSA = O-specific antigen. Representative top agar assays (of at least three independent replicates) underlying the interpretation shown in this illustration are shown in panel (**b**) and Fig. S6. **b** Top agars were set up with *P. aeruginosa* PAO1 *Δpel Δpsl* (wild type) and different engineered or spontaneously isolated mutants lacking functional expression of one or more surface receptor genes before infection with serial dilutions of phage Paride and control phages E79 (targeting the LPS core^83^), newly isolated phage Victoria (targeting the LPS O-antigen), or DMS3*vir* (targeting type IV pili^89^). Arrows highlight opaque plaque formation of phage Paride on several mutants. Strain EM-095 is a spontaneously isolated mutant with a single nucleotide deletion that leads to inactivation of the *ssg* gene. Strain EM-307 is a spontaneously isolated “brown mutant” as described previously with a large deletion around *galU*^49^. The data are summarized in Table S2.

Phage PA5oct had previously been shown to have a partial requirement for type IV pili and lipopolysaccharides (LPS) of its *P. aeruginosa* host^46^ which are both very common—though usually distinct—phage receptors on this organism^47,48^. We readily confirmed that Paride infectivity is partially compromised in absence of either type IV pili (Δ*pilA*), O-antigen (Δ*wbpL*; Fig. 4a, b), or flagella (Δ*fliC*, Fig. S6). Using a panel of spontaneously resistant mutants, we determined that hosts with deeper truncations of the LPS core below the O-antigen (Δ*galU* or Δ*ssg*) are completely and not only partially resistant to Paride (Fig. 4a, b). These genes had already previously been implicated in resistance to LPS-targeting phages infecting *P. aeruginosa*^48–50^. Based on these results, we conclude that the essential terminal receptor for Paride infections is located in the outer core of the *P. aeruginosa* LPS and probably includes its $\alpha$-glucose(III) moiety (Figs. 4a and S6; see also in “Methods” and Table S2)^51–53^.

### Phage-antibiotic synergy of Paride and meropenem sterilizes deep-dormant cultures in vitro and reduces bacterial loads in vivo

**Fig. 5:**
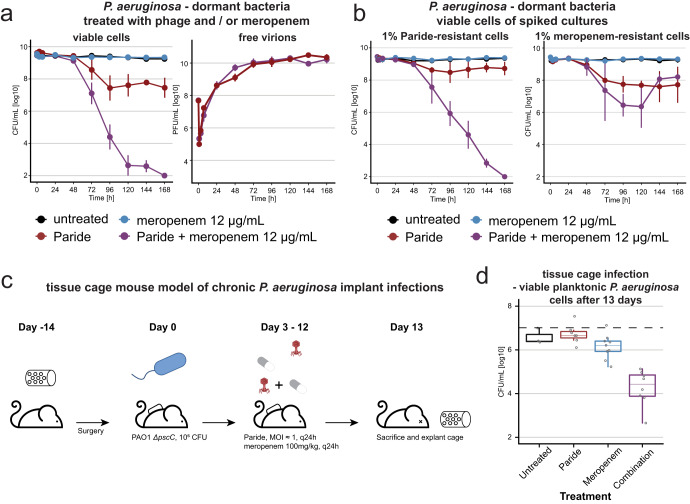
Paride-meropenem synergy eradicates bacteria in vitro and reduces bacterial loads in vivo. **a** *P. aeruginosa* PAO1 *Δpel Δpsl* subcultured for 48 h were treated with meropenem alone or in combination with Paride (MOI ≈ 0.01) and viable CFU/ml as well as free phages were recorded over time. The red kill curve (Paride) is the same as in Fig. 2b and shown here again for comparison. Analogous experiments with control phages, ciprofloxacin, and tobramycin are shown in Fig. S7a–c. **b** *P. aeruginosa* PAO1 *Δpel Δpsl* subcultured for 48 h and spiked with 1% of either Paride- or meropenem-resistant cells of the same growth state (see Table S3) were treated with Paride (MOI ≈ 0.01), meropenem or the combination thereof and viable CFU/ml as well as free phages (Fig. S7d) were recorded over time. Data points and error bars in (a,b) show the average of three independent experiments and their standard error of the mean. Limits of detection are 2 log10 CFU/mL for viable cells and 3.6 log10 PFU/mL for free phages. **c** Schematic representation of murine tissue cage infection experiments (see “Methods”). **d** Boxplots showing the viable planktonic bacteria recovered from the tissue cage at the end of the tissue cage infection (see Fig. S7E for the data of each mouse and time point and Fig. S7F for the analogous data of adherent bacteria). Each dot represents the surviving bacteria recovered from one mouse of either three (untreated) or six (each treatment condition) examined across two independent experiments. Boxplots visualize the median, two hinges, two whiskers and any outliers beyond the defined ranges. The hinges represent the 25th and 75th percentile respectively, while the whiskers extend from the respective hinge on a value no further than 1.5 times the interquartile range from the hinge (where IQR is the interquartile range, or distance between the first and third quartiles). Any data beyond this distance are outliers. For transparency in reporting, we have displayed all data points individually, including outliers. The dashed line represents the median initial inoculum at the start of treatment. The limit of detection is 1.6 log10 CFU/mL. Source data are provided as a Source data file.

The combined treatment of bacterial infections with antibiotic drugs and bacteriophages can have a strong synergistic effect, but these interactions are difficult to predict and mostly applied empirically^54^. We therefore investigated whether the combination of Paride with antibiotic drugs might enable the killing of more than the ca. 99% of deep-dormant cells that are eliminated by the phage alone before a plateau of phenotypic resistance is reached (see Fig. 2b). For this purpose, the Paride infection experiments of deep-dormant cultures were repeated in combination with lethal concentrations of the fluoroquinolone ciprofloxacin, the aminoglycoside tobramycin, or the carbapenem meropenem. Treatment with meropenem and Paride together resulted in complete sterilization of deep-dormant *P. aeruginosa* cultures in vitro to the detection limit even though meropenem alone had no detectable effect under these conditions^40^ (Figs. 5a and S7a). Conversely, combining phages with ciprofloxacin or tobramycin had no effect beyond the bactericidal action of the antibiotics alone (Fig. S7b, c), probably because lethal concentrations of these drugs inhibit central dogma processes required for phage replication. To gain further insight into the nature of the phage-antibiotic synergy of Paride and meropenem, we repeated the experiment by spiking deep-dormant cultures of wildtype *P. aeruginosa* with 1% of phage- or meropenem-resistant bacteria (Figs. 5b and S7d, Table S3). In cultures spiked with phage-resistant bacteria the outcome of the experiment was unchanged, while the addition of meropenem-resistant bacteria largely abolished the synergy (compare Fig. 5a, b). This suggests that the phage-antibiotic synergy is caused by antibiotic killing of bacteria that have been sensitized to the drug by the phage-induced lysis of bystanders and not vice versa.

Given this striking in vitro phenotype, we then explored if the phage-meropenem synergy could also be observed in vivo. We therefore adapted the previously established murine tissue cage model^55^ to simulate chronic implant infections of *P. aeruginosa* (Fig. 5c). Briefly, mice were surgically implanted with a Teflon cage on their back which was subsequently infected with *P. aeruginosa*. Three days later, a daily treatment with Paride, meropenem, or the combination of both was started and continued up to thirteen days post-infection (Fig. 5c, see “Methods”). Strikingly, while neither phage nor drug treatment alone had any strong effect, the combination of both greatly reduced planktonic bacteria by ca. 3 logs and (more modestly) adherent bacteria inside the tissue cage, confirming the showed Paride-meropenem synergy also in vivo (Figs. 5d and S7e, f).

### Productive infection of deep-dormant hosts by Paride requires functional stress responses

**Fig. 6:**
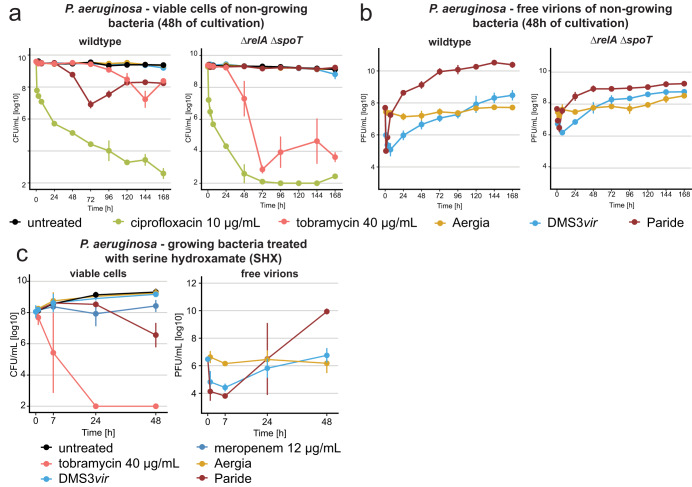
Paride requires stress responses for infection of dormant hosts. **a**, **b** Deep-dormant cultures of *P. aeruginosa* PAO1 *Δpel Δpsl* (wildtype) and its *ΔrelA ΔspoT* derivative both grown in M9Rich were treated with antibiotics or phages (MOI ≈ 0.01) and viable CFU/ml as well as free phages were recorded over time. **c** Growing cultures of *P. aeruginosa* were treated with 1 mM of DL-serine hydroxamate for 12 h and then challenged with antibiotics or phages (MOI ≈ 0.01). Viable CFU/ml as well as free phages were recorded over time (see Fig. S8e for a control experiment without SHX). Data points and error bars show the average of three independent experiments and their standard error of the mean. Limits of detection are 2 log10 CFU/mL for viable cells and 3.6 log10 PFU/mL for free phages. Source data are provided as a Source data file.

Given the correlation of antibiotic tolerance and resilience to phage infections for deep-dormant bacteria (compare Fig. 1 and Figs. 1–3)^40^, we hypothesized that the bacterial core signaling orchestrating their dormant physiology might be responsible for both phenomena. In many Gram-negatives, the stringent response second messenger (p)ppGpp and stress response sigma factor RpoS together tune down cellular processes in stationary phase which is thought to cause antibiotic tolerance^6–8^. We therefore tested whether knocking out the makers and breakers of (p)ppGpp (*relA* and *spoT*) or the stress response sigma factor *rpoS* might sensitize non-growing *P. aeruginosa* to phages other than Paride. As expected, the *ΔrpoS* and *ΔrelA ΔspoT* mutants displayed greatly reduced antibiotic tolerance in a non-growing state after 48 h of cultivation (Figs. 6a and S8a). However, there was no clear difference between these mutants and the parental wildtype during rapid growth (Fig. S8b–d; in line with previous work on (p)ppGpp and tolerance of *E. coli*^56^). These results confirm that (p)ppGpp and RpoS signaling primarily contribute to dormancy and antibiotic tolerance after cells have entered stationary phase.

Intriguingly, the *ΔrpoS* and *ΔrelA ΔspoT* mutants were still not more permissive to infection by control phages when grown into growth arrest and instead even became highly refractory to infection by Paride under these conditions (Figs. 6a, b and S8a–d). Notably, Paride infections of regularly growing *ΔrpoS* or *ΔrelA ΔspoT* strains were indistinguishable from the parental wildtype (Fig. S8b–d). These results suggest that the ability of Paride to directly replicate on non-growing hosts depends on subversion of the regular stationary phase physiology of deep-dormant bacteria. Subsequently, we performed the inverse experiment and caused strong starvation-like signaling in originally growing cultures using the stringent response inducer serine hydroxamate (SHX) (Fig. 6c)^9^. As expected, SHX treatment induced considerable antibiotic tolerance and general resilience to phage infection with only Paride maintaining its ability to replicate (Figs. 6c and S8e).

## Discussion

### Bacteriophages use at least two different strategies to infect dormant host cells

Our study confirms previous notion that bacteriophages are generally unable to directly replicate on deep-dormant bacteria^24,25^, but presents a new *P. aeruginosa* phage named Paride with the unique ability to kill deep-dormant bacteria by direct lytic replication (Figs. 1 and 2b). Notably, no *E. coli* phage with this ability could be isolated despite considerable efforts. In line with the literature on this organism, we speculate that *P. aeruginosa* as an environmental generalist may have a more active stationary phase that enables higher vigilance but also causes a higher sensitivity to ciprofloxacin treatment (Fig. 1e) and, possibly, to phage infections. Interestingly, efficient replication of Paride on growth-arrested hosts specifically requires cellular stress responses in form of (p)ppGpp and RpoS signaling that are dispensable for infections of growing hosts (Figs. 6a, b and S8a–d). These results indicate that Paride subverts certain aspects of the host’s dormant physiology to enable direct replication, e.g., by mobilizing resources and energy that are stored away in regular stationary phase cells and might not be available in growth-arrested hosts lacking the core stress and starvation responses^6,7^. Since RpoS and (p)ppGpp are not significantly contributing to the physiology of growing cells^6,7^, our results suggest a significant functional plasticity of phage Paride and possibly different infection strategies for growing and dormant host cells.

Unlike Paride, most other phages enter a more or less stable state of hibernation in deep-dormant host cells (Fig. 1c, d). Previous work on phage T4 hibernation showed that it hinges on the arrest of its lytic program in dormant cells after degrading the host chromosome and in dependence on lysis control gene *rI*^28,57^. This would suggest that hibernation can be a phage-imposed strategy activated after irreversible takeover of a dormant cell to postpone replication until more resources are available to maximize viral productivity. Such hibernation thus truly represents a form of “pseudolysogeny” in which the virus seeks shelter from UV radiation and other environmental hazards inside bacterial cells^30^. In these cases, the infected cells carrying hibernating phages will certainly die at latest upon resuscitation when the phage completes its lytic cycle. While this has previously occasionally been interpreted as viral killing of dormant, antibiotic-tolerant bacteria^58^, neither the replication of phage particles nor the death of the host cell occur during dormancy (unlike for Paride). This distinction is ecologically important because it separates two very different infection strategies either prioritizing fast reproduction (direct replication like Paride) or a possibly higher burst size (hibernating phages) analogous to the decisions between lysis and lysogeny of temperate phages. It also matters for phage therapy in vivo because only direct replication but not phage hibernation would support immediate local amplification of the virus at the infection site.

It will be interesting to see how general this “virus-imposed hibernation” is as a phage strategy compared to alternative scenarios such as, e.g., a host-imposed viral paralysis due to resource limitation. The latter hypothesis would interpret dormancy as a physiological defense against phage infection, though different from classical abortive infection that shuts down critical host processes at the cost of cellular survival to suffocate viral spread through the population^59^. Recent work indeed showed that host dormancy strongly promotes the acquisition of CRISPR-Cas immunity against infecting phages^60^ and enhances the potency of a restriction-modification system^61^. It is well imaginable that infection strategies like the one of Paride and of T7 which enable direct replication on dormant hosts might have evolved at least in part to counter such physiological defenses against viral infections.

### Different results obtained with different experimental models for “stationary phase”

This work was largely performed using our previously described experimental setup for studying the biology of deep-dormant bacteria that is based on defined culture media and rigorously controlled assay conditions^40^. These technical details matter because experimentation with antibiotic-tolerant, dormant bacteria is notoriously sensitive to seemingly small changes in the assay setup^10,12,38,39^. The deep-dormant cells in our experiments have been in a non-growing state under severe nutrient limitation for ca. 40h^40^. They are stably dormant without loss of cell viability and are non-dividing because we do not observe significant killing even under prolonged treatment with lethal concentrations of β-lactam drugs that poison bacterial cell wall biosynthesis, eliminating any intermittently growing cell (Figs. 2c, 5a, and S7e)^40,62^. This is different from other methodologies sometimes used in the field. In particular, cultures grown to stationary phase in LB broth were shown to exhibit significant spontaneous cell death, an equilibrium of growing and dying cells, and considerable killing by β-lactams^63,64^.

When evaluated over time from early stationary phase to deep dormancy 48 h after subculturing, our non-growing bacteria are changing physiologically (see, e.g., the proteomic analyses at different time points; Fig. S1) and, intuitively, seem to become progressively more dormant as evidenced by increasing antibiotic tolerance (compare the data in Fig. 1 with those in Figs. 2a and S1–3). These physiological changes over time have direct biological impact. As an example, we could reproduce the previously reported ability of phage T7 to replicate on stationary phase hosts only in early stationary phase (up to 8 h after subculturing, but not anymore after 12 h; compare Fig. 2a to S2c).

Similarly, we readily reproduced previously published results on the hibernation of *E. coli* phage T4 and *P. aeruginosa* phage UT1^28,29^ in dormant hosts (Fig. 1c, d). Conversely, we failed to identify a stationary phase condition where phages T4 and UT1 could robustly replicate (Fig. 1c, d as well as S2 and S3), in contrast to previously published results^28,35^. These differences could be due to the very high MOIs where T4 replication on stationary phase bacteria had been observed or caused by the specific environmental *P. aeruginosa* strain grown in lake water where UT1 replication in starved *P. aeruginosa* had been seen.

### Phage-antibiotic synergy of Paride and meropenem sterilizes in vitro cultures and reduces bacterial loads in vivo

One of the most exciting results of our study is that a combination of Paride and meropenem can sterilize deep-dormant cultures in vitro (Fig. 5a) and greatly reduce a resilient bacterial infection of a tissue cage implant in mice (Fig. 5c, d and S7e, f). Notably, Paride alone can kill only around 99% of cells in deep-dormant cultures (Figs. 2b and 5a) while meropenem alone or in combination with other phages is completely ineffective (Fig. 5a, d and S7a–c)^40^. A key difference between our results in vivo and in vitro is that Paride treatment alone failed to cause detectable killing of bacteria residing in the mouse tissue cage (Fig. 5d) despite significant efficacy in vitro (Fig. 2b). This discrepancy might be caused by the dense and complex setup in vivo which may inhibit phage activity in different ways, e.g., through local immune responses or by physically restricting viral access to some bacterial cells.

The experiments performed by spiking dormant cultures with dormant phage- or drug-resistant bacteria (Fig. 5b) suggest that the observed strong phage-antibiotic synergy is a chain reaction initiated by lysis of some deep-dormant cells by Paride. Molecules released from these cells might cause resuscitation of phenotypically phage-resistant bystanders and enable their effective killing by meropenem as soon as cell wall biosynthesis resumes and can be poisoned by β-lactams^62^. This resuscitation might be caused by nutrients released by phage-mediated lysis^65^ or by cell wall fragments and possibly other signaling molecules as resuscitation signals for dormant cells^3^. Understanding this phage-antibiotic synergy on the molecular level might enable us to design new treatment options for resilient bacterial infections based on the forced resuscitation of deep-dormant, drug-tolerant bacteria.

## Methods

Our research complies with all relevant ethical regulations and good practice in the field. Animal experiments were performed in accordance with Swiss federal regulations and the license (permit number 1710) was approved by the cantonal veterinary office of Basel-Stadt (Switzerland).

### Preparation of culture media and solutions

Lysogeny Broth (LB) was prepared by dissolving 10 g/L tryptone, 5 g/L yeast extract, and 10 g/L sodium chloride in Milli-Q H_2_O and sterilized by autoclaving. LB agar plates were prepared by supplementing LB medium with agar at 1.5% w/v before autoclaving. M9Glc was prepared as described previously^40^. The M9Rich culture medium was conceived as a variant of regular M9Glc medium supplemented with 10% v/v LB medium (prepared without NaCl) to promote the growth of diverse strains^40^. It was prepared from sterilized components by mixing (for 50 mL) 33.75 mL Milli-Q H2O, 10 mL 5× M9 salts solution, 5 mL LB medium without NaCl, 500 μl 40% w/v D-glucose solution, 100 μL 1 M MgSO_4_, and 5 μL 1 M CaCl2 using sterile technique. Unless indicated otherwise, all components were sterilized by filtration (0.22 μm). Phosphate-buffered saline (PBS) was prepared as a solution containing 8 g/L NaCl, 0.2 g/L KCl, 1.44 g/L Na_2_HPO_4_x2H_2_O, and 0.24 g/L KH_2_PO_4_ with the pH adjusted to 7.4 using 10 M NaOH and sterilized by autoclaving. SM buffer was prepared as 0.1 M NaCl, 10 mM MgSO_4_, and 0.05 M Tris (pH 7.5) using sterile technique.

### Bacterial handling and culturing

*E. coli* and *P. aeruginosa* strains were routinely cultured in LB medium at 37 °C in glass culture tubes or Erlenmeyer flasks with agitation at 170 rpm. For all antibiotic treatment and phage infections assays, the bacteria were instead grown in M9Glc or M9Rich. Clinical isolates of *P. aeruginosa* often showed fastidious growth requirements and were always cultivated in M9Rich. LB agar plates were routinely used as solid medium. Selection for genetic modifications or plasmid maintenance was performed with gentamicin at 20 μg/mL, ampicillin 100 μg/mL, oxytetracycline 12.5 μg/mL for *E. coli* or gentamicin at 30 μg/mL, carbenicillin 100 μg/mL, and oxytetracycline 100 μg/mL for *P. aeruginosa*.

### Bacteriophage handling and culturing

Bacteriophages (listed in Table S1) were generally cultured using the double-agar overlay (“top agar”) method with a top agar prepared as LB agar with only 0.5% w/v agar supplemented with 20 mM MgSO_4_ and 5 mM CaCl_2_^66,67^. Top agar plates were incubated at 37 °C for ca 16 h before plaque enumeration, with the exception of phage T7 for which plaques were enumerated after ca. 3 h of incubation (before they grew too large in size). High-titer stocks of bacteriophages were generated using the plate overlay method. Briefly, top agar plates were set up to grow almost confluent plaques of a given phage and then covered with 12 mL of SM buffer. After careful agitation for 24–72 h at 4 °C, the suspension on each plate was pipetted off and centrifuged at 8000 × *g* for 10 min. Supernatants were sterilized with few drops of chloroform and stored in the dark at 4 °C.

For in vivo use, phage particles were purified by layered cesium chloride gradient ultra-centrifugation similar to previous work^68^. Briefly, a 10 ml sample of phage stock was loaded on top of a 9 ml gradient of six steps (from *ρ* = 1.2 g/cm^3^ to *ρ* = 1.7 g/cm^3^) and then centrifuged at 78,200 × *g* for 18 h at 20 °C. Subsequently, a clearly visible light blue phage band was harvested with a syringe. The collected sample with a final volume of ca. 2–3 ml was dialyzed at 4 °C in SM buffer.

### Bacterial strains and strain construction

All bacterial strains used in this work are listed in Table S3. The *P. aeruginosa* phage isolation strain *P. aeruginosa* PAO1 *hsdR17* was generated by two-step allelic exchange using suicide plasmid pEX18-Tc with suitable homology regions^69^. All remaining mutants were generated using pFOGG-based suicide plasmids (see Table S5). Plasmids were either electroporated (2.5 kV/25 µF/400 Ω) or mated into their host using *E. coli* JKE201 as donor strain^70^.

### Plasmid construction

Plasmids were commonly constructed using classical restriction-ligation cloning or the method of Gibson et al. (“Gibson Assembly”)^71^ by ligating PCR products guided by 25 nt overlaps. Point mutations in plasmids were introduced by PCR with partially overlapping primers using the method of Liu and Naismith^72^. *E. coli* strain EC100 *pir(+)* was the host strain of al molecular cloning and successful plasmid construction was routinely assessed by Sanger Sequencing. All oligonucleotide primers used in this study are listed in Table S4 and all plasmids are listed in Table S5. The construction of all plasmids is described in Supplementary Data 1.

### Bacteriophage isolation

Bacteriophages described in this study were isolated between March 2019 and March 2021 generally using ZnCl_2_ precipitation of source samples as described previously^73^, and a complete list of all used phages can be found in Table S1. To isolate phages infecting bacteria in stationary phase, we used 10 ml of deep-dormant culture of *E. coli* K-12 MG1655 or *P. aeruginosa* PAO1 *hsdR17* as described previously and then added 50–300 μL of phage precipitate^40^. Upon addition, a 100 µl aliquot was plated by double agar overlay and used to estimate the number of phages initially present. Upon agitation in Erlenmeyer flasks for 48–168 h at 37 °C, the cultures were centrifuged at full speed for 5 min and the supernatants transferred to fresh tubes. Supernatants were sterilized with a few drops of chloroform before 100 µl were plated by double agar overlay and the rest was stored at 4 °C. We then counted plaques after overnight incubation at 37 °C to evaluate whether phage replication had occurred during the cultivation on the deep-dormant culture. Phage isolates with the ability to replicate on dormant hosts were propagated and stocked following standard procedures^73^.

### Antibiotic treatment and phage infection assays

Time-resolved kill curves with phages and antibiotics were generally performed as described previously (see also Fig. 1a)^40^. Briefly, bacterial cultures were challenged with phages or antibiotics either directly after subculturing (to target growing bacteria) or at different times afterwards (typically 48 h for deep dormancy). Samples were withdrawn from these cultures over time to track bacterial survival and phage infections. Viable cell counts were determined by plating serial dilutions of samples that had been washed in PBS (to remove residual antibiotics or free virions) on LB agar plates. In addition to determining viable cell counts, we also recorded the free phage titer and the number of infected cells whenever appropriate. Free phages (i.e., free virions in the culture) were sampled by plating serial dilutions of the culture supernatant on top agar plates of a suitable host strain (as described by Bryan and colleagues^28^). The number of infected cells was determined by spotting the samples from the serial dilutions used for the viable cell quantification onto a top agar plate of the respective host bacterium. In the absence of free virions, plaques originate from infected bacteria as centres of infection. Colony forming units (CFU) and plaque-forming units (PFU) were typically recorded after 16–24 h of incubation at 37 °C after which no appearance of additional colonies or plaques has been observed. Unless indicated differently, these experiments were performed using a *Δpel Δpsl* knockout of *P. aeruginosa* PAO1 that lacks functional expression of the Pel and Psl exopolysaccharides to reduce the formation of biofilms during long-time cultivation that can greatly distort the results of liquid culture experiments as described previously^39,40^.

The experiment shown in Fig. 6c was performed by growing a culture of *P. aeruginosa Δpel Δpsl* into stationary phase for 36 h and then diluting it back 1:10 into fresh medium containing 1 mM of DL-serine hydroxamate (which arrested bacterial growth) before incubation for 12 h at 37 °C with continued agitation. Subsequently, antibiotic and phage treatment were started, viable cells and free phages were sampled and quantified as usual. As control, a parallel experiment (Fig. S8e) with a culture freshly diluted 1:10 into fresh medium was performed analogously.

### Bacteriophage genome sequencing, assembly, and annotation

Bacteriophage genomes were purified using the Norgen Biotek Phage DNA Isolation Kit and sequenced at the former Microbial Genome Sequencing Center (MiGS) using Illumina Technology. Genome assembly and downstream analyses were performed using Geneious Prime 2021.0.1 following standard procedures in the field^73^. Phage genomes were annotated using Pharokka v1.3.0^74^ followed by manual curation. Coding sequences (CDS) were predicted with PHANOTATE v1.5.1^75^ and tRNAs were predicted with tRNAscan-SE v2.0.11^76^.

### Sequence alignments and phylogenetic analyses

For the phylogeny shown in Fig. 2d, the major capsid protein, terminase large subunit, and DNA polymerase amino acid sequences were extracted from several phages belonging to group 2.2 of jumbo phages as defined by Iyer et al.^42^ and distantly related myoviruses T4 (NCBI GenBank accession NC_000866.4) and Cr30 (NCBI GenBank accession NC_025422.1) as outgroup. Besides Paride and its closely related isolates described in this study, we included *Agrobacterium* phage Atu_ph07 (NCBI GenBank accession NC_042013.1), *Escherichia* phage PBECO4 (NCBI GenBank accession NC_027364.1), *Salmonella* phage Munch (NCBI GenBank accession MK268344.1)), and *Xanthomonas* phage XacN1 (NCBI GenBank accession AP018399.1). The phylogeny was generated following standard procedures in the field as described previously for other bacteriophages^73^. Briefly, amino acid sequences were aligned using MAFFT v7.450^77^ implemented in Geneious Prime 2021.0.1, manually curated, and then concatenated to calculate a Maximum-Likelihood phylogeny using PhyML 3.3.20180621^78^ implemented in Geneious Prime 2021.0.1.

### Morphological analyses by transmission electron microscopy

The virion morphology of Paride was analyzed by transmission electron microscopy following common procedures in the field^79^. Briefly, 5 μl drops of high-titer lysate were adsorbed to 400 mesh carbon-coated grids, which were rendered hydrophilic using a glow-discharger at low vacuum conditions. They were subsequently stained on 5 μl drops of 2% (w/v) uranyl acetate. Samples were examined using an FEI Tecnai G2 Spirit transmission electron microscope (FEI Company, Hillsboro, Oregon, USA) operating at 80-kV accelerating voltage. Images were recorded with a side-mounted Olympus Veleta CCD camera 4k using EMSIS RADIUS software at a nominal magnification of typically ×150,000.

### Clinical isolate selection and infection

Clinical isolates of *P. aeruginosa* from cystic fibrosis patients were generously shared by the University Hospital of Basel via Prof. Urs Jenal (Supplementary Data 2). Candidates for testing of Paride susceptibility were chosen randomly with preference for high-tolerance isolates described in the study by Santi, Manfredi, and colleagues^80^. We first screened a total of 91 *P. aeruginosa* isolates for general susceptibility to Paride (with 21/91 being susceptible) and then selected ten isolates for stationary phase infections based on robust growth in M9Rich and LB agar top agars. We determined the MIC of ciprofloxacin and tobramycin for the relevant *P. aeruginosa* strains as described before^40^ in M9Rich (Table S8). These strains were then grown to late stationary phase like in regular Paride infection experiments (see above) and infected with Paride at an MOI of ca. 1:5000. Free phage titers were determined after 48 h of cultivation of 37 °C and compared to the inoculum to detect possible phage replication (Fig. 3a).

### Lipopolysaccharides and bacteriophage surface receptors on *P. aeruginosa* PAO1

To gain further insight into the essential host receptor of Paride, we isolated spontaneously resistant mutants by plating bacteria on LB agar plates which had been densely covered with high-titer lysates of the phage. After whole-genome sequencing, we determined the efficiency of plating for several phages with different known receptors on these mutants (Table S2, Figs. 4 and S6). Through the comparison of the EOP, the known structures of different receptor mutants (*ΔwbpL* and *ΔgalU*) and proposed phenotypes for PA5001 (*ssg*) from previous studies, we concluded that the secondary receptor of phage Paride is likely to be at the α-glucose(III) moiety of the core LPS (Fig. 4). Since the exact structure of the LPS formed by a *P. aeruginosa* PAO1 *ssg* (PA5001) mutant is unknown, we highlighted the sugar suspected to be missing by crossing it off in red (Fig. 4). The remaining residues were represented with dashed lines to indicate that their presence is uncertain. The image is not drawn to scale and was adapted and redrawn from different sources^51–53,81–83^.

### Bacterial genome sequencing and assembly

For bacterial whole-genome sequencing, genomic DNA was prepared using the GenElute Bacterial Genomic DNA Kit (Sigma-Aldrich, St. Louis, Missouri, USA) according to the manufacturer’s guidelines and sequenced at the former Microbial Genome Sequencing Center (MiGS) using the Illumina NextSeq 550 platform. Genome assembly and mutation mapping were performed using *breseq* (https://github.com/barricklab/breseq)^84^.

### Experimental evolution of Paride by passaging on stationary phase cultures

Two parallel cultures of *P. aeruginosa* were grown to stationary phase as described previously^40^ and at the start infected with Paride at an MOI of 1:100,000. Infected cultures (5 ml volume) were agitated at 37 °C for 72 h (first 40 transfers) which was later shortened to 24 h (transfers 41 to 71). At each transfer, a sample of each previous infection culture was sterilized with chloroform and diluted 1:100,000 into a freshly grown stationary phase culture. At the end of the experimental evolution, single plaques were picked from both evolutionary lines and used for further experimentation as Paride_1 and Paride_2. After 40, 55, and 71 transfers (corresponding to ca. 340, 470, and finally 600 generations) we sequenced the genome of single-plaque isolates from both lines (see Fig. S5).

### Quantification of Paride infections using one-step growth curves

One-step growth curve experiments were designed based on established procedures in the field^85,86^. Bacteria were first grown from in M9Glc medium from single colony for 24 h at 37 °C and subsequently diluted back 1:100 for additional 24 h of cultivation. Fast-growing cultures were generated by an additional 1:100 dilution of this dense culture followed by 3 h of cultivation at 37 °C shaking. Subsequently, 1 ml of culture was spun down at maximal speed in a tabletop centrifuge and resuspended in 100 μl of fresh M9Glc medium (obtaining ca. 10^9^ CFU/ml). For stationary phase experiments, 1 ml of the original dense culture was used.

Cells with phage at an MOI of ca. 0.1 followed by 15 min of adsorption at 37 °C shaking before the sample was diluted 1:10,000 into 25 mL of pre-warmed medium to prevent further infection cycles. While regularly growing cells were diluted into M9Glc, stationary phase bacteria were diluted back into M9nocarbon, a variant of M9Rich medium where no carbon source and no LB broth are added, to prevent resuscitation when encountering fresh medium. These cultures were agitated in Erlenmeyer flasks using a shaking water bath at 37 °C (Julabo SW22). We measured the number of initially infected cells and changes in free phage titers over time by double-layer agar assays as described above.

The latency period was determined as the first timepoint where extracellular phages could be detected among at least two technical replicates. Burst size was estimated by dividing the average number of free phages at the plateau of PFU formation by the number of infected cells upon dilution.

### Efficiency of plating experiments

The infectivity of a phage on a given host was quantified by determining the efficiency of plating (EOP), i.e., by quantifying its plaque formation on this host in comparison to plaque formation on reference strain *P. aeruginosa* PAO1 *Δpel Δpsl* following standard procedures in the field^73^.

### Proteomics sample preparation

Cultures were grown for 24 h in M9Glc from −80 °C cryostocks. Subsequently, they were diluted back 1:100 into fresh medium pre-warmed to room temperature. At 3, 12, 24, and 48 h post-dilution the equivalent of 1 mL at OD600 0.6 (corresponding to ca. 5 × 10^8^ CFU/mL) was collected, spun down (10,000 × *g*, 2 min), supernatant was removed, pellets were flash frozen in liquid nitrogen and stored at −80 °C. The whole proteomes of PAO1 *ΔpelΔpsl* and MG1655 were determined following the Standard Operative Procedures (SOP v.2020.09.03) at the Proteomic Core Facility of the Biozentrum, University of Basel (Switzerland). A detailed description of the laboratory and the analysis procedures is accessible in a dedicated methodologic tutorial article^87^.

### Proteomics analyses

Heatmaps, principal component analysis (PCA), and clustering analyses were performed using the R packages pheatmap, Stats, and dtwclust, respectively. Graphics were generated with the ggplot2 R package. Proteomics “raw.” files and corresponding metadata are accessible in the MassIVE dataset MSV000091557 (http://massive.ucsd.edu).

### Tissue cage infection experiments

The murine tissue cage model closely resembles human infections and is well established for research primarily on the persistence of *Staphylococcus aureus*^55,88^. Briefly, this model is based on subcutaneous insertion of cylindric tissue cages followed by experimental infection of the foreign body by injection of bacterial inoculum into the lumen of the cages. For our study, we adapted this system to mimic persistent implant infections with *P. aeruginosa*. These experiments used a partially attenuated mutant of *P. aeruginosa* PAO1 lacking functional type III secretion (Δ*pscC*) because the wild type caused systemic infection and death of infected mice within 48 h. All work was performed according to the regulations of Swiss veterinary law (#1710) in the animal facility of the Department of Biomedicine, University Hospital Basel (Switzerland). Mice were housed in a 12-h light/dark cycle (light from 7 am to 7 pm) in a temperature-controlled room (24 °C) at 45% (+/−10%) humidity with free access to regular mice chow and water. For tissue cage experiments, each one sterile polytetrafluorethylene (Teflon) cylinder (32 × 10 mm), perforated by 130 regularly spaced holes of 1 mm diameter (tissue cages; Angst-Pfister AG, Zürich) was aseptically implanted subcutaneously into the back of a 13-week-old female C57BL/6 mouse (minimum weight 20 g; obtained from Janvier Labs (France)). Experiments were started after complete wound healing (minimum 2 weeks after surgery). The cylindric tissue cages were infected with 1.16 × 10^5^ CFU of *P. aeruginosa* PAO1 Δ*pscC*. Three days post-infection, the mice were randomly assigned to one of the following experimental groups: untreated (*n* = 3), phage Paride (10^7^ PFU, directly injected into the cylinder, qdam; *n* = 6), meropenem (Labatec, Switzerland; 100 mg/kg, i.p., qdam; *n* = 6), or a combination of both (*n* = 6). Phage Paride was administered at a calculated MOI = 1 based on the bacterial load as determined on day 2 post inoculation by aspiration and tissue cage fluid (TCF) plating. Over the treatment time of 10 days the planktonic bacterial load was recorded by plating serial dilutions on agar plates (day 4, day 7, day 11 post-infection). On day 13 post-infection, TCF was aspirated, mice were sacrificed, and the tissue cages were explanted under aseptic conditions. Explanted tissue cages were washed twice with PBS followed by 30 s vortexing, sonication for 3 min at 130 W, and finally again 30 s vortexing to release adherent bacteria from the biofilm. Quantification of adherent bacteria as CFUs was performed by plating serial dilutions on agar plates and enumeration of bacterial colonies after overnight incubation at 37 °C.

### Quantification and analysis

Quantitative data sets were analyzed by calculating mean and standard error of the mean of independent biological replicates for each experiment. Detailed information about replicates and statistical analyses for each experiment is provided in the figure legends and the Source data file. Data were analyzed in Microsoft Excel and plotted using R-Studio and the ggplot2 package.

### Reporting summary

Further information on research design is available in the Nature Portfolio Reporting Summary linked to this article.

### Supplementary information

Supplementary Information

Peer Review File - NEW

Description of Additional Supplementary Files

Supplementary Dataset 1

Supplementary Dataset 2

Reporting Summary

### Source data

Source Data File

## Data Availability

Source data of the experiments presented in this study are included as a Source data file. Genome sequences of all newly isolated and sequenced phages have been deposited in the NCBI GenBank repository. The genome of *Pseudomonas* phage Aergia has been deposited with accession OR805291, the genome of *Pseudomonas* phage Cassandra has been deposited with accession OR805292, the genome of *Pseudomonas* phage Deifobo has been deposited with accession OR805293, the genome of *Pseudomonas* phage Ettore has been deposited with accession OR805294, the genome of *Pseudomonas* phage Paride has been deposited with accession OR805295, and the genome of *Pseudomonas* phage Victoria has been deposited with accession OR805296. Raw data of all proteomics experiments have been deposited in the ProteomeXchange database and the MassIVE repository under accession codes PXD041131 and MSV000091557, respectively. Source data are provided with this paper.

## Code availability

The R-studio code used for the analysis of CFU and PFU curve data is available at the Zenodo repository with the digital object identifier 10.5281/zenodo.10063932
