# Supplementary material for: Phage Paride can kill dormant, antibiotic-tolerant cells of Pseudomonas aeruginosa by direct lytic replication
Source: Nat Commun. 2024 Jan 2;15:175. doi: 10.1038/s41467-023-44157-3 (PMC10761892; doi:10.1038/s41467-023-44157-3)
Supplement: Supplementary file 1 — Supplementary Information [file 41467_2023_44157_MOESM1_ESM.pdf]

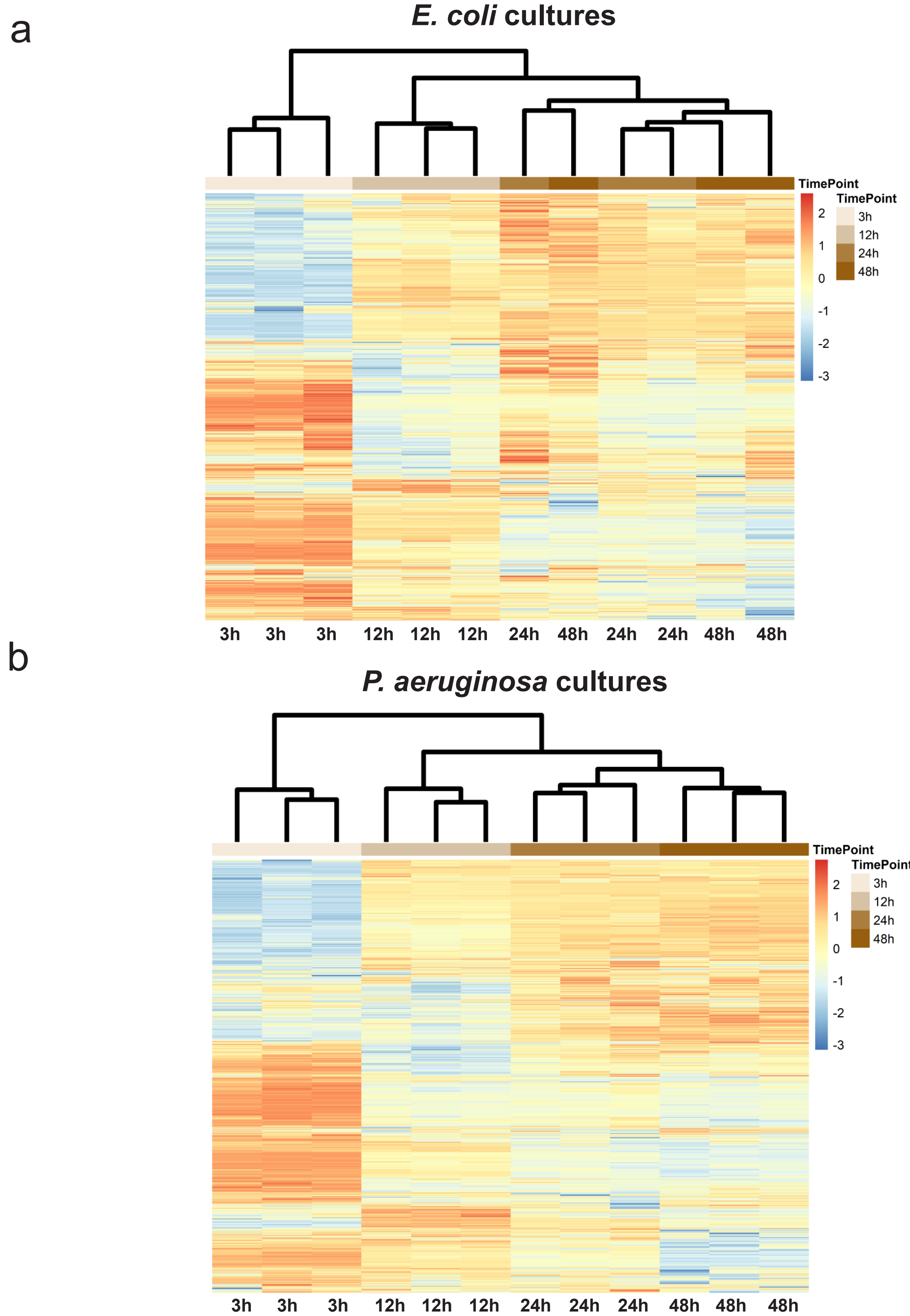

Figure S1: **Whole proteomes of *E. coli* and *P. aeruginosa* at various growth stages.**
Heatmaps of the total proteomes of *E. coli* K-12 MG1655 **(a)** and *P. aeruginosa* Δ*pel* Δ*psl* **(b)** sampled at 3, 12, 24, and 48h of growth in M9Glc. The dendrograms (top) indicate how the proteomes from different time points cluster together. Colors (blue to red) on the heatmaps indicate the log2 intensity of the mean-centered MS signals for each protein detected. The data indicate a clear progression of the proteomic profiles from the earliest (light brown, growing bacteria) to the latest time point (dark brown; deep dormancy 48h after subculturing). In this culture setup, the bacteria reach stationary phase (maximal optical density at 600nm wavelength) after ca. eight hours as shown in our previous work[1]. All data are available in public repositories (see *Data Availability*).

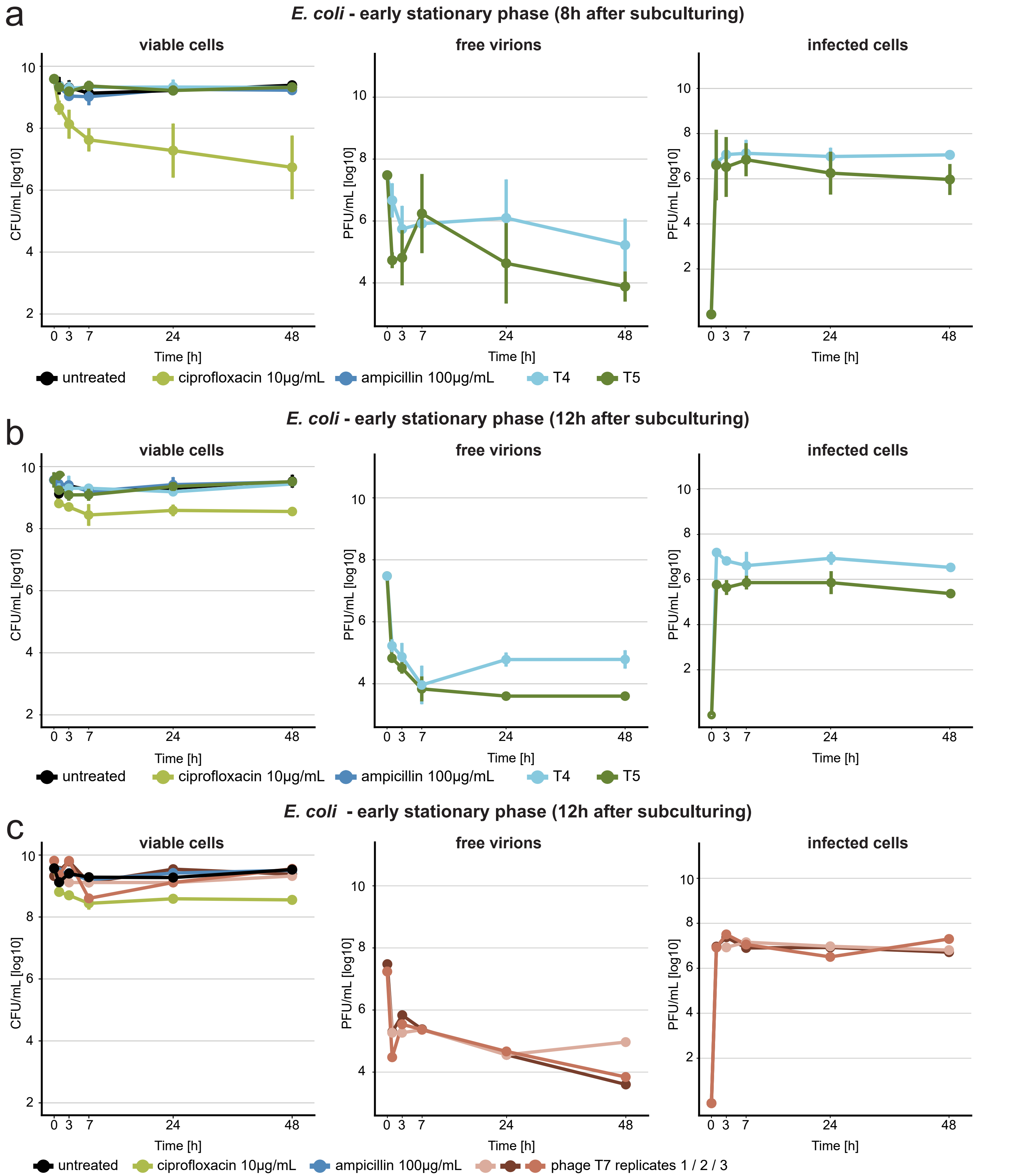

Figure S2: **Time-kill curves of early stationary phase *E. coli*.**

(a-c) *E. coli* K-12 MG1655 cultured for 8h or 12h after subculturing were challenged with antibiotics or phages (MOI ≈ 0.01) and viable cells (CFU/ml) as well as plaque-forming units (PFU/ml) of free virions and infected cells were recorded over time. Data points and error bars show the average of three biological replicates and standard error of the mean, with the exception of (c) where the individual replicates of the experiments performed with phage T7 are shown (matching Fig. 2a). Limits of detection are 2 log10 CFU/mL for viable cells, 3.6 log10 PFU/mL for free virions and 2.6 log10 PFU/mL for infected cells. Source data are provided as a Source Data file.

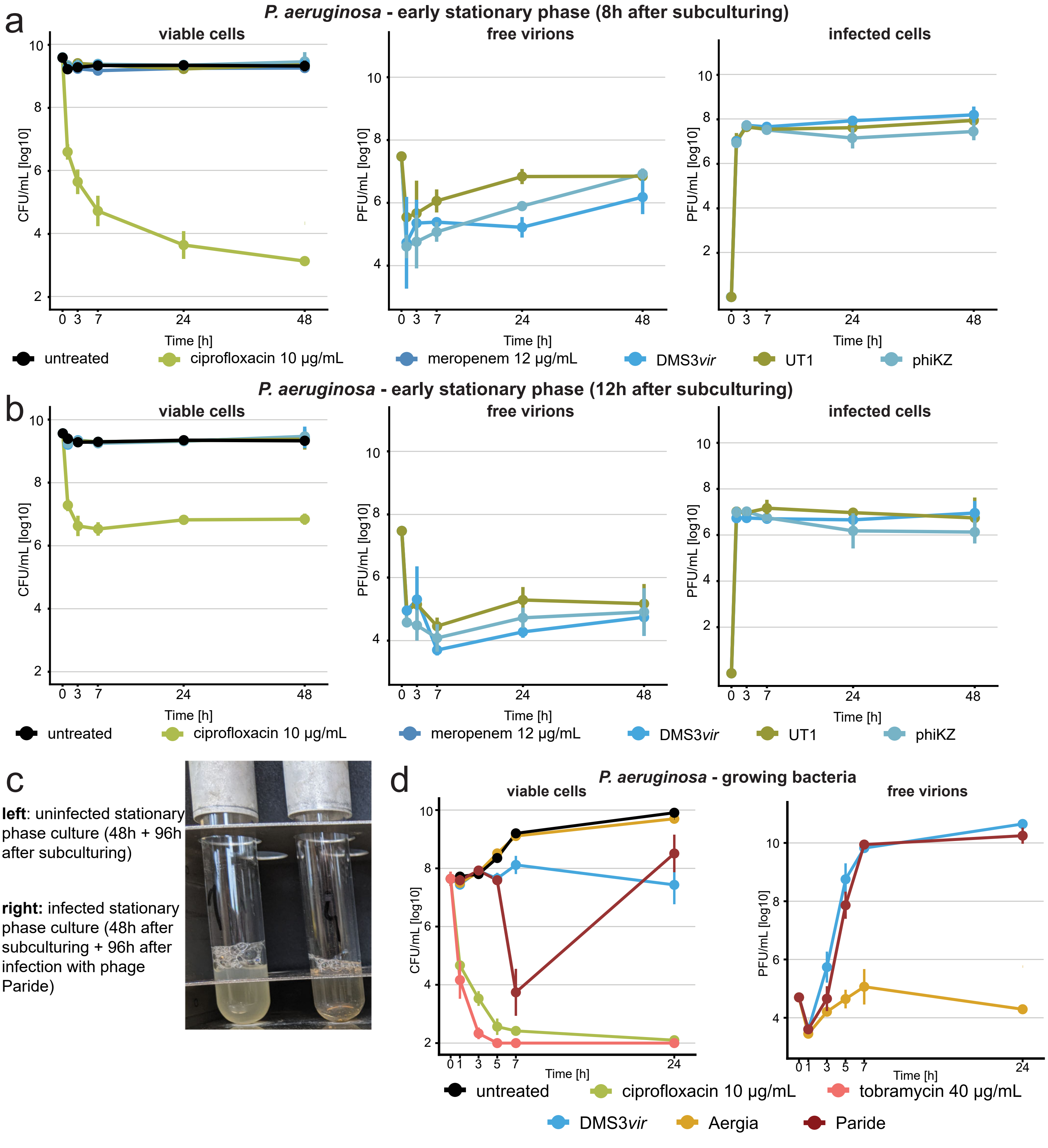

Figure S3: **Time-kill curves of early stationary phase *P. aeruginosa*, infection of regularly growing *P. aeruginosa* with Paride and lysis of deep-dormant cultures by the same phage.**
(a-b) *P. aeruginosa* Δ*pel* Δ*psl* cultured for 8 or 12h after subculturing were challenged with antibiotics or phages (MOI ≈ 0.01) and viable cells (CFU/ml) as well as plaque-forming units (PFU/ml) of free virions and infected cells were recorded over time. **(c)** Representative picture of a deep-dormant *P. aeruginosa* culture at 96h after infection with Paride in M9Glc (right) and untreated control (left). **(d)** Regularly growing cultures of *P. aeruginosa* were treated with antibiotics or phages (MOI ≈ 0.001) and viable CFU/ml as well as free phages were recorded over time. Data points represent the average of three independent experiments and error bars show their standard error of the mean. Limits of detection are 2 log10 CFU/mL for viable cells, 3.6 log10 PFU/mL for free phages and 2.6 log10 PFU/mL for infected cells. Source data are provided as a Source Data file.

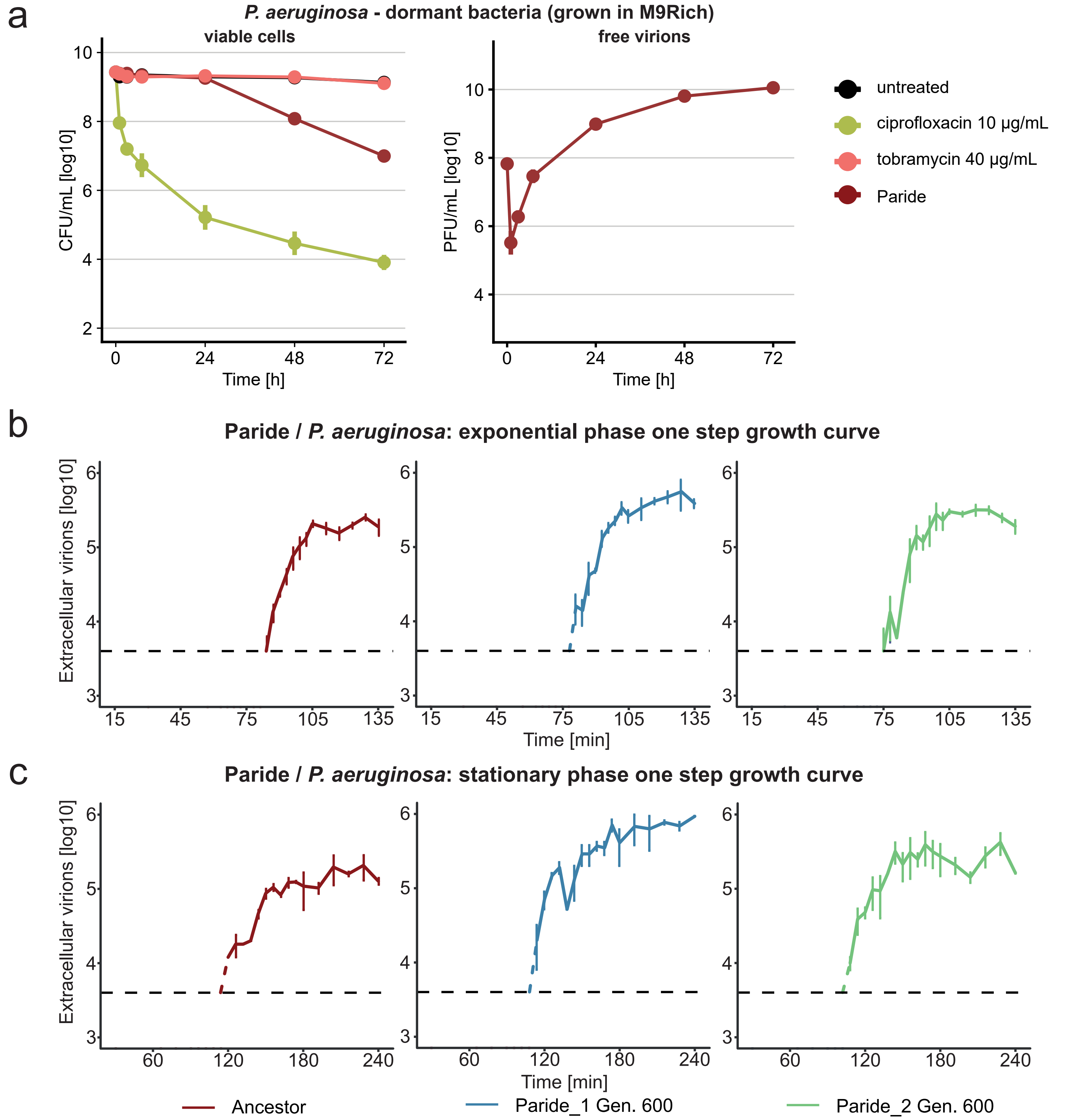

Figure S4: **Stationary phase time-kill curves of *P. aeruginosa* in M9Rich and one step growth curves of Paride.**

(a) Deep-dormant cultures of *P. aeruginosa* Δ*pel* Δ*psl* grown in M9Rich were treated with antibiotics or phages (MOI ≈ 0.01) and viable CFU/ml as well as free phages were recorded over time. Data points represent the average of three independent experiments and error bars show their standard error of the mean. Limits of detection are 2 log10 CFU/mL for viable cells, 3.6 log10 PFU/mL for free virions and 2.6 log10 PFU/mL for infected cells. **(b, c)** Free virions of one-step growth experiments with ancestral Paride and two lineages evolved on deep-dormant cultures for ca. 600 generations were recorded over time in fast-growing cultures (b) and stationary phase cultures (c). Data points represent the average of six (regularly growing) and two (stationary phase) independent experiments, respectively. The dashed line represents the limit of detection (3.6 log10 PFU/mL). Source data are provided as a Source Data file.

a

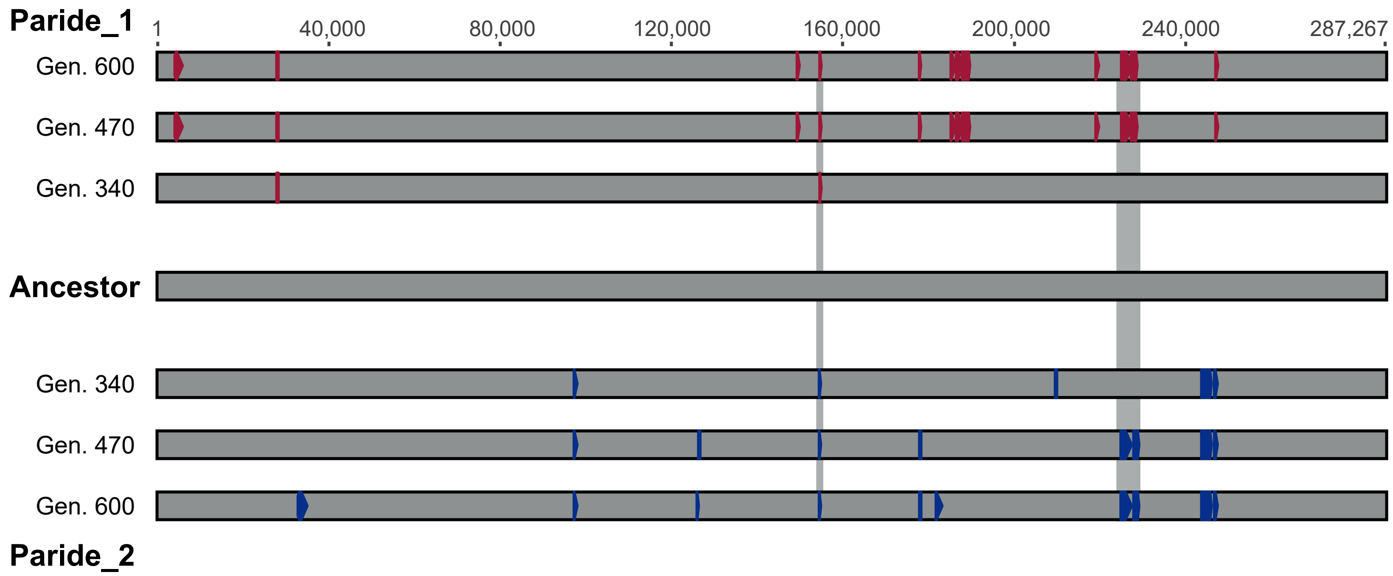

b

Location (Ancestor)	Annotation	Mutation	Potential Identity/Function	First observation	
				Paride_1	Paride_2
5'379	Portal (connector) protein	Synonymous SNP C→T	Portal connector or vertex protein	470 gen.	-
28'409	Intergenic space	G→A	-	340 gen.	-
33'455	rIIA lysis inhibitor	Single bp deleted → ~90% truncated	rIIA protein	-	600 gen.
97'875	FtsH-interacting integral membrane protein	Non-synonymous SNP C→T (Thr→Ile)	-	-	340 gen.
126'471	Structural protein	Non-coding SNPs (470 gen.) Deletion (502 bp; 600 gen.)	Collar protein	-	600 gen.
150'220	Hypothetical protein	Single bp insertion → frameshift alters a few residues and truncates by 6	Isomerase or oxidoreductase, aligns to UDP-glucose-4-epimerases	470 gen.	-
155'004	Intergenic space directly upstream of hypothetical protein	SNP A→G (ATA ATG.. to ATG ATG..)	-	340 gen.	340 gen.
178'493	Hypothetical protein	Synonymous SNP A→G	-	470 gen.	-
178'650	Intergenic space upstream of hypothetical protein	A→G	-	-	470 gen.
183'289	ATPase	Non-synonymous SNP T→C (Ser→Pro)	AAA+ ATPase / protease	-	600 gen.
186'457	Multiple genes without specific annotation	Deletion (3'277bp)	-	470 gen.	-
210'329	Intergenic region	Deletion of duplicated region (67bp)	-	-	Only 340 gen.
219'823	Structural protein	Non-synonymous SNP C→A (Gln→Lys)	-	470 gen.	-
225'313	Multiple genes	Deletion (3'758bp)	-	470 gen.	-
225'398		Deletion (4'209bp)		-	470 gen.
244'531	Multiple genes without specific annotation	Deletion (4'284bp)	-	-	340 gen.
247'901	Hypothetical Protein	Synonymous SNP C→T	-	470 gen.	-

Figure S5: **Comparative genomics of evolved Paride lineages and mutation analysis.**
(a) Schematic genome alignment of Paride_1 and Paride_2 lineages from clones sampled at different time points (after ca. 340, 470, and 600 generations of evolution). Grey bars highlight spots where the same or similar mutations have been detected. **(b)** Full list of mutations identified in the two evolved Paride lineages. Possible biological functions of mutated genes have been inferred from the genome annotation. The table also indicates the first detection of each mutation with respect to the three clones that have been sequenced sequentially for each lineage. All data are shown directly in this illustration.

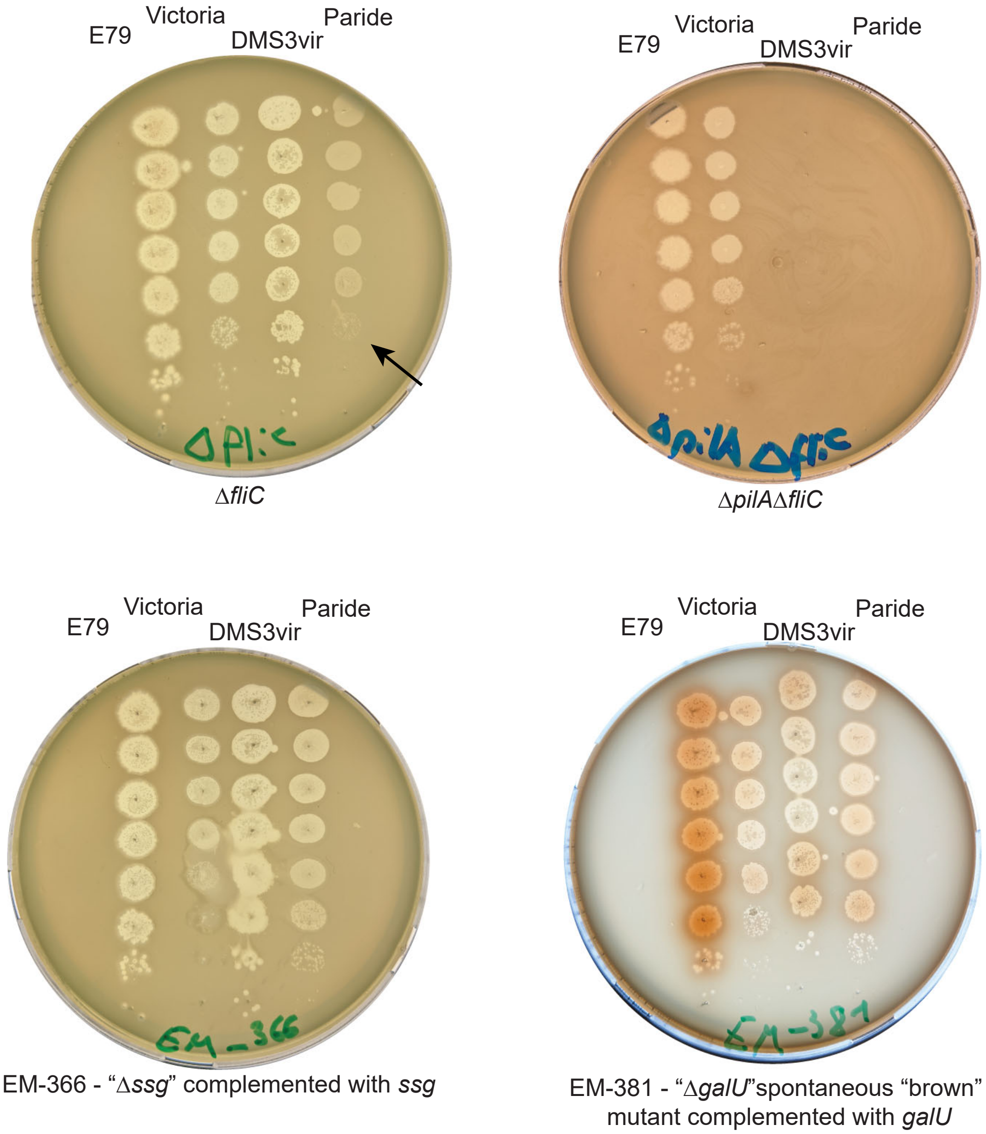

Figure S6: **Top agar assays with Paride and control phages on different surface receptor mutants of *P. aeruginosa* PAO1.**
Top agars were set up with *P. aeruginosa* PAO1 Δ*pel* Δ*psl* mutants lacking functional expression of one or more surface receptor genes before infection with serial dilutions of phage Paride and control phages E79 (targeting the LPS core; see Meadow & Wells, *Microbiology* (1978)[2]), newly isolated phage Victoria (targeting the LPS O antigen), and DMS3*vir* (targeting type IV pili; see Budzik et al., *J Bacteriol* (2004)[3]). Arrows highlight opaque plaque formation of phage Paride on several mutants. Strain EM-366 is is a spontaneously isolated mutant with a single nucleotide deletion which inactivates the *ssg* gene complemented with the same gene *in cis*. Strain EM-381 is a spontaneously isolated "brown mutant" as described previously with a large deletion around *galU* (Markwitz et al., *ISME J* (2022)[4]) complemented with *galU in cis*. The data are summarized in Table S2. Top agar plates are representatives of at least three independent replicates.

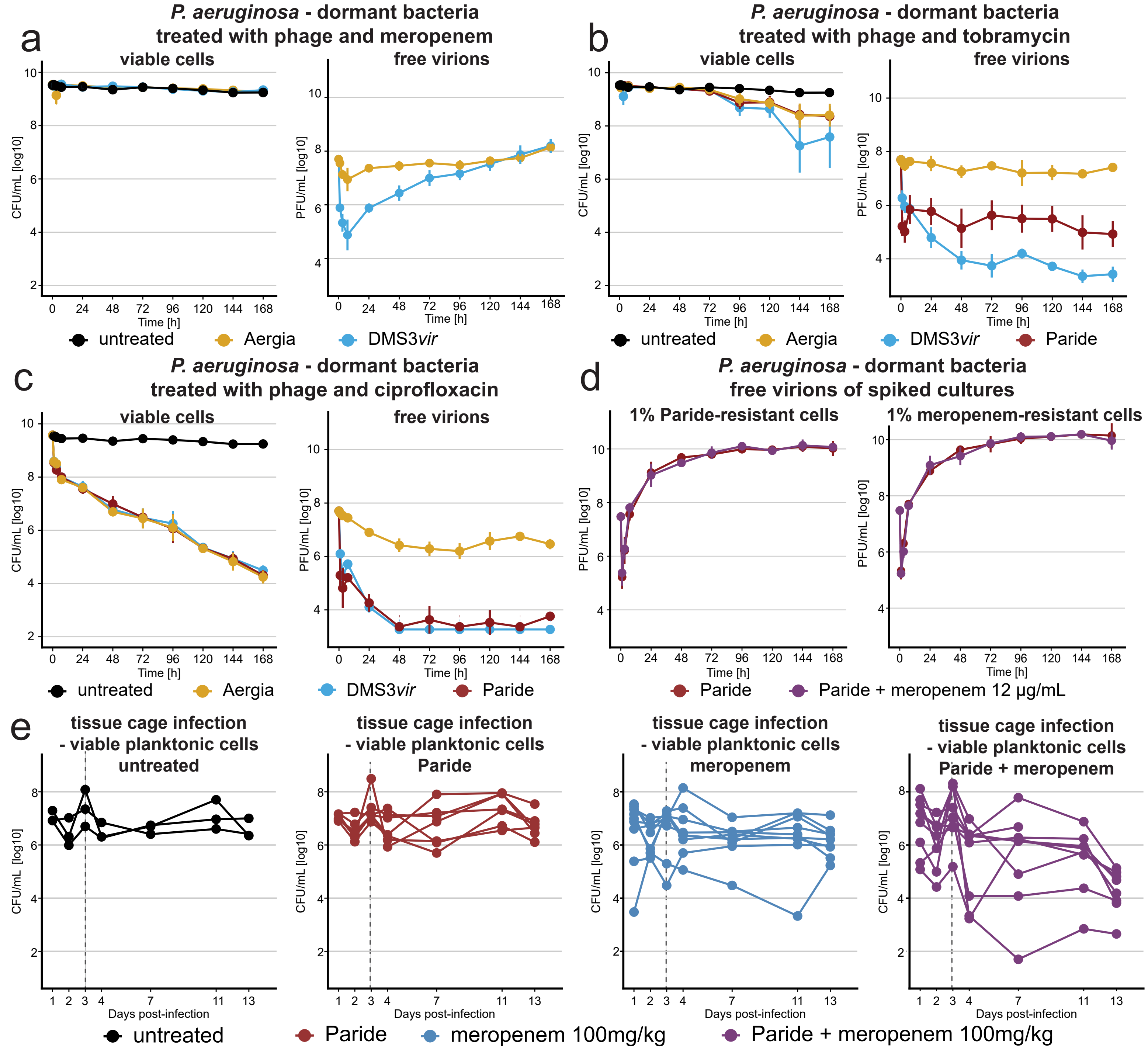

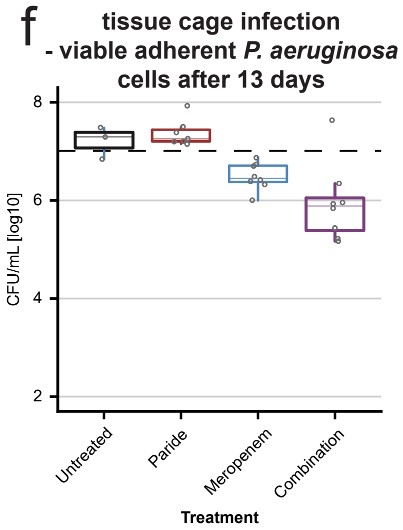

Figure S7: **Additional data of phage-antibiotic combination treatments and tissue cage infections experiments.**
(a-c) Deep-dormant cultures of *P. aeruginosa* were treated with meropenem (12 µg/ml), tobramycin (40 µg/ml) or ciprofloxacin (10 µg/ml) in combination with phages (MOI ≈ 0.01) and viable CFU/ml as well as free phages were recorded over time. **(d)** *P. aeruginosa* was cultured for 48h after subculturing and spiked with 1% of either Paride- or meropenem resistant cells from analogous cultures before treatment with Paride (MOI ≈ 0.01), meropenem, or the combination thereof and viable CFU/ml (Fig. 5) as well as free phages were recorded over time. Data points and error bars show the average of three independent experiments and their standard error of the mean. Limits of detection are 2 log10 CFU/ml for viable cells and 3.6 log10 PFU/ml for free phages. **(e)** The planktonic bacterial load of the tissue cage infection experiments was determined over time and plotted per mouse grouped by treatment condition (dashed line = treatment start). Each data point represents the CFU/ml recovered from tissue cage fluid of one mouse. **(f)** Boxplots analogous to Fig. 5d showing viable adherent bacteria recovered from tissue cages at the end of the experiment (see *Methods*). Each dot represents the surviving bacteria recovered from one mouse and the dashed line shows the median initial inoculum at the treatment start. The limit of detection is 1.6 log10 CFU/ml. Source data are provided as a Source Data file.

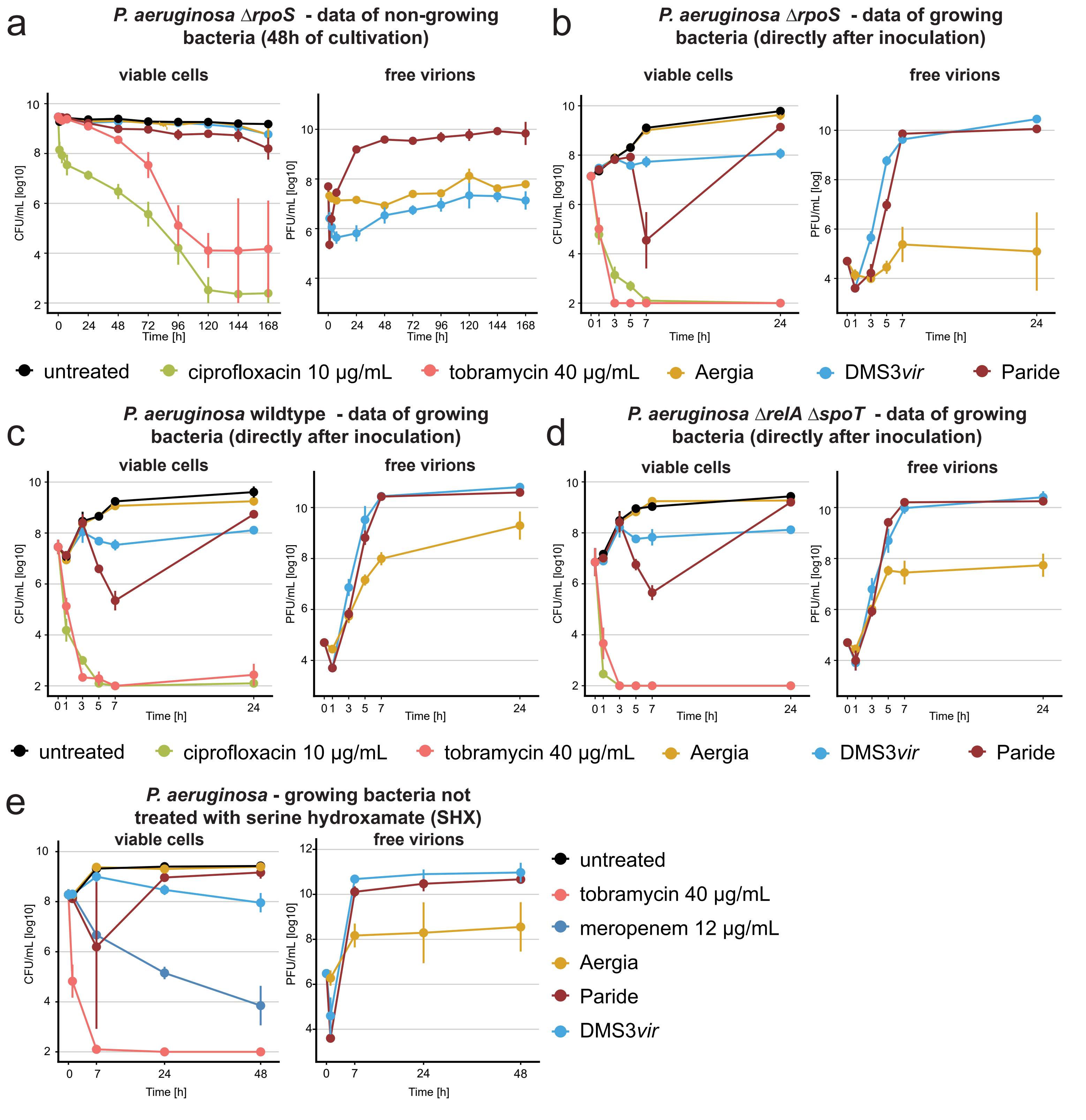

Figure S8: **Additional data regarding Paride infections of Δ*rpoS* and Δ*relA* Δ*spoT* mutants as well as in presence of SHX.**
(a) Deep-dormant cultures of *P. aeruginosa* PAO1 Δ*pel* Δ*psl* (wildtype) and its Δ*rpoS* derivative both grown in M9Glc were treated with antibiotics or phages (MOI ≈ 0.01) and viable CFU/ml as well as free phages were recorded over time. **(b)** Fast-growing cultures of *P. aeruginosa* PAO1 Δ*pel* Δ*psl* (wildtype) and its Δ*rpoS* derivative both grown in M9Glc were treated with antibiotics or phages (MOI ≈ 0.001) and viable CFU/ml as well as free phages were recorded over time. **(c,d)** Fast-growing cultures of *P. aeruginosa* PAO1 Δ*pel* Δ*psl* (wildtype) and its Δ*relA* Δ*spoT* derivative both grown in M9Rich were treated with antibiotics or phages (MOI ≈ 0.001) and viable CFU/ml as well as free phages were recorded over time. **(e)** Growing cultures of *P. aeruginosa* were processed as described for the SHX treatment shown in Fig. 6c (as a control without SHX) and then challenged with antibiotics or phages (MOI ≈ 0.01). Viable CFU/ml as well as free phages were recorded over time. Data points and error bars show the average of three biological replicates and standard error of the mean. Limits of detection are 2 log10 CFU/mL for viable cells, 3.6 log10 PFU/mL for free phages. Source data are provided as a Source Data file.

Table S1 – Bacteriophages used in this study

**Common name**	**Systematic name**	**Host**	**Isolation source**	**Reference**
T4	vB_EcoM_T4	*E. coli* K-12 MG1655	NA	our laboratory collection
Bas37	vB_EcoM_KarlGJung	*E. coli* K-12 MG1655	NA	Maffei, Shaidullina, et al., *PLoS Biology* (2021)[5]
T5	vB_EcoS_T5	*E. coli* K-12 MG1655	NA	our laboratory collection
Bas27	vB_EcoS_TrudiGerster	*E. coli* K-12 MG1655	NA	Maffei, Shaidullina, et al., *PLoS Biology* (2021)[5]
T7	vB_EcoP_T7	*E. coli* K-12 MG1655	NA	our laboratory collection
Bas07	vB_EcoS_JakobBernoulli	*E. coli* K-12 MG1655	NA	Maffei, Shaidullina, et al., *PLoS Biology* (2021)[5]
Bas08	vB_EcoS_DanielBernoulli	*E. coli* K-12 MG1655	NA	Maffei, Shaidullina, et al., *PLoS Biology* (2021)[5]
Bas54	vB_EcoM_MaxBurger	*E. coli* K-12 MG1655	NA	Maffei, Shaidullina, et al., *PLoS Biology* (2021)[5]
Bas60	vB_EcoM_PaulScherrer	*E. coli* K-12 MG1655	NA	Maffei, Shaidullina, et al., *PLoS Biology* (2021)[5]
DMS3vir	vB_PaeS_DMS3vir	*P. aeruginosa* PAO1	NA	Budzik et al., *J Bacteriol* (2004)[3]
Aergia	vB_PaeS_Aergia	*P. aeruginosa* PAO1	marsh sample (Sursee, CH; March 2019)	this study
Victoria	vB_PaeM_Victoria	*P. aeruginosa* PAO1	sewage inflow (ARA Canius, Lenzerheide, CH; April 2019)	this study
UT1	vB_PaeM_UT1	*P. aeruginosa* PAO1	NA	Schrader et al., *Can J Microbiol* (1997)[6] ; obtained from Prof. Tyler Kokjohn
phiKZ	vB_PaeM_phiKZ	*P. aeruginosa* PAO1	NA	Krylov and Zhazykov, *Genetika* (1978)[7]; obtained from the Felix d'Herelle Reference Center for Bacterial Viruses

Paride	vB_PaeM_Paride	*P. aeruginosa* PAO1	rotting plant material from Hörnli cemetery (Basel, CH; May 2019)	this study
Ettore	vB_PaeM_Ettore	*P. aeruginosa* PAO1	sewage inflow (ARA Basel, CH; March 2020)	this study
Deifobo	vB_PaeM_Deifobo	*P. aeruginosa* PAO1	sewage inflow (ARA Basel, CH; October 2020)	this study
Cassandra	vB_PaeM_Cassandra	*P. aeruginosa* PAO1	sewage inflow (ARA Basel, CH; February 2021)	this study

Table S2 – Phage susceptibility of *P. aeruginosa* PAO1 surface receptor mutants

(+) – clear plaques, (+/-) opaque plaque, (-) reduced efficiency of plating, (--) loss of lytic activity

**Strain**	**Phenotype**	**E79 activity**	**Victoria activity**	**DMS3vir activity**	**Paride activity**
PAO1	PAO1 wildtype strain	(+)	(+)	(+)	(+)
PAO1 *ΔpelΔpsl*	wildtype strain of this study	(+)	(+)	(+)	(+)
PAO1 *ΔpilA*	lack of type IV pili	(+)	(+)	(--)	(+/-)
PAO1 *ΔfliC*	lack of flagella	(+)	(+)	(+)	(+/-)
PAO1 *ΔpelΔpsl ΔpilA ΔfliC*	lack of type IV pili and lack of flagella	(+)	(+)	(--)	(--)
PAO1 *ΔpelΔpsl ΔwpbL*	lack of glucosyltransferase WbpL essential for the initiation of O-antigen synthesis	(+)	(--)	(+)	(+/-)
PAO1 *ΔpelΔpsl ΔgalU*	lack of uridylyltransferase GalU essential for the synthesis of LPS and O-antigen precursors	(--)	(--)	(+)	(--)
PAO1 *ΔpelΔpsl Δssg*	lack of glucosyltransferase Ssg (PA5001) essential for the synthesis of O-antigen	(--)	(--)	(+)	(--)
PAO1 *ΔpelΔpsl* "EM-095"	spontaneous mutant resistant to Paride with a (GGC → G-C deletion at position 5'618'713 that causes a frameshift in *ssg* (PA5001)	(--)	(--)	(+)	(--)
PAO1 *ΔpelΔpsl* "EM-307"	spontaneous mutant with "brown" phenotype resistant to Paride; carries a deletion of 44'015bp between positions 2'198'124-2'230'850 and 2'231'737-2'242'134 encompassing *galU*	(--)	(--)	(+)	(--)
PAO1 *ΔpelΔpsl* "EM-366"	EM-095 complemented with SC101miniTn7-Gm-Plac_PA5001 (*ssg*)	(+)	(+)	(+)	(+)
PAO1 *ΔpelΔpsl* "EM-381"	EM-307 complemented with SC101miniTn7-Gm-Plac_*galU*	(+)	(+)	(+)	(+)

Phage E79 is known to depend on structures in the LPS core of *P. aeruginosa* PAO1 (Meadow and Wells, *Microbiology* (1978)[2]) while phage DMS3*vir* targets type IV pili (Budzik et al., *J Bacteriol*

(2004)[3]). Newly isolated phage Victoria requires expression of the LPS O-antigen of *P. aeruginosa* PAO1 as evidenced by loss of lytic activity on a *wbpL* knockout. Base pair coordinates refer to the *P. aeruginosa* PAO1 reference genome (NCBI GenBank accession AE004091).

Table S3 - Strains used in this study

**Strains**	**Description**	**Reference**
E. coli K-12 MG1655	laboratory wildtype strain of *E. coli*	our laboratory collection
P. aeruginosa PAO1	parental strain of all *P. aeruginosa* PAO1 strains used in this study	our laboratory collection
PAO1 *hsdR17*	phage isolation strain of *P. aeruginosa* PAO1 where the *hsdR* component of the *hsdRMS* system was inactivated; the strain is otherwise of wildtype genotype with functional *pel* and *psl* loci	this study
PAO1 *ΔpelΔpsl*	strain with reduced biofilm formation, used as wildtype strain for all experiments unless stated otherwise	Broder et al., *Nat Microbiol* (2016)[8]
PAO1 *ΔpelΔpsl ΔrelAΔspoT*	strain lacking the (p)ppGpp producing and degrading enzymes RelA and SpoT	this study
PAO1 *ΔpelΔpsl ΔrpoS*	strain lacking the RpoS sigma factor	this study
PAO1 *ΔpelΔpsl ΔwbpL*	strain lacking the glycosyltransferase WbpL essential for O-antigen biosynthesis initiation	this study
PAO1 *ΔpelΔpsl ΔgalU*	strain lacking the uridylyltransferase GalU essential for the biosynthesis of LPS and O-antigen precursors	this study
PAO1 *ΔpilA*	strain lacking the major pilin PilA, unable to assemble type IV pili	Laventie et al., *Cell Host Microbe* (2019)[9]
PAO1 *ΔfliC*	strain lacking the flagellin FliC, unable to assemble flagella	Laventie et al., *Cell Host Microbe* (2019)[9]
PAO1 *ΔpelΔpsl ΔpilA ΔfliC*	strain lacking PilA and FliC, unable to assemble pili and flagella	Jenal lab collection
PAO1 *ΔpelΔpsl* "EM-095"	spontaneous mutant resistant to Paride carrying a point deletion at position 5'618'713 (GGC →G-C) in the PA5001 gene causing a frameshift; this gene codes for Ssg, an enzyme involved in LPS core biosynthesis (see *Methods*), strongly suggesting that this structure is part of the host receptor used by Paride; this mutant was isolated as a survivor of a deep-dormancy infection	this study
PAO1 *ΔpelΔpsl* "EM-307"	spontaneous mutant with "brown" phenotype resistant to Paride carrying a deletion of 44'015bp between positions 2'198'124-2'230'850 and 2'231'737-2'242'134 around *galU*	this study
PAO1 *ΔpelΔpsl* "EM-366"	EM-095 complemented with SC101miniTn7-Gm-Plac_PA5001/*ssg*	this study
PAO1 *ΔpelΔpsl* "EM-381"	EM-307 complemented with SC101miniTn7-Gm-Plac_*galU*	this study
PAO1 *ΔpelΔpsl* "EM-429"	wildtype strain transformed with mCTX::ev	this study

PAO1 *ΔpelΔpsl* "EM-430"	wildtype strain transformed with pUC18miniTn7::ev	this study
PAO1 *ΔpelΔpsl* "EM-431"	"EM-095" transformed with mCTX::ev	this study
401190-12 – Pseaer "EM-460"	clinical isolate used as template for the generation of EM-497	this study
PAO1 *ΔpelΔpsl oprD_CI NDM* "EM-497"	strain engineered to have high meropenem resistance (see Table S7); it carries an inactivating mutation in the *oprD* gene (involved in meropenem import) and was transformed with SC101miniTn7 encoding a constitutively expressed NDM β-lactamase amplified from EM-460;	this study
PAO1 *ΔpscC* "EM-358"	strain lacking PscC, an essential structural component of the type III secretion system	this study

Base pair coordinates refer to the *P. aeruginosa* PAO1 reference genome (NCBI GenBank accession AE004091).

Table S4 – Oligonucleotide primers used in this study

**Primers**	**Description**	**Sequence 5'->3'**
prAH2057	Linearisation of pFOGG vector	CATAAAGTGTAAAGCCTGGGG
prAH2058	Linearisation of pFOGG vector	GCTATCTGGACAAGGGAAAAC
prAH2079	Shuttling primers from pEX18-Tc to pFOGG	TTGCGTTTTCCCTTGTCCAGATAGCCCAGTCACGACG TTGTAAAAC
prAH2283	Shuttling primers from pEX18-Tc to pFOGG	GGCACCCCAGGCTTTACACTTTATGCAGGAAACAGC TATGACCATG
prEM0028	To amplify *hsdR* insert	CGCCAT**TCTAGA**CTCATCGAAGCCGGTGACGAATT
prEM0029	To amplify *hsdR* insert	CGCCAG**GAGCTC**AACTCGACAATAAGCCGGGCAAG
prEM0005	To introduce M353(ATG->TGA) stop mutation in *hsdR* sequence	TGCGGTCACAAAGGGTCGGCTACGCTG
prEM0006	To introduce M353(ATG->TGA) stop mutation in *hsdR* sequence	CTTTGTGACCGCAGTTCGATTCCATCATC
prEM0013	Verification of *hsdR17* mutation	ATCGCTGGCCAACATCATCG
prEM0014	Verification of *hsdR17* mutation	GTGCGCCTCGTCGATCAATA
prEM0135	Verification of *relA* deletion	GTGACTGGCAACTGACTCTGG
prEM0136	Verification of *relA* deletion	GATCGACCTTGAGATGCCG
prEM0070	Verification of *spoT* deletion	GTCTTCGGCAACCTCTACGGCA
prEM0071	Verification of *spoT* deletion	GTCGTCGCCATAGAAGGCAACC
prEM0072	Verification of *rpoS* deletion	AGTTAGTACGTCGGTACCTGC
prEM0073	Verification of *rpoS* deletion	AACATCACCGAGAAGAAGGA

prEM0312	To amplify *galU* deletion insert	TTGCGTTTTCCCTTGTCCAGATAGCCTACTCCTGGATCAGATGCGT
prEM0313	To amplify *galU* deletion insert	CTCATGATCAAGAAGGCTCACTGAGCCTCGCC
prEM0314	To amplify *galU* deletion insert	CCTTCTTGATCATGAGAATCCTTCGACA
prEM0315	To amplify *galU* deletion insert	GGCACCCCAGGCTTTACACTTTATGTCGCCACCAAGATTTCCTTCA
prEM0316	Verification of *galU* deletion	CGATGACGAACAGATCGAAATC
prEM0317	Verification of *galU* deletion	AGTTCAGCAAGTACGCGGC
prEM0059	To amplify *wbpL* deletion insert	TTGCGTTTTCCCTTGTCCAGATAGCGTTCCACCAGTTGGGCTTG
prEM0060	To amplify *wbpL* deletion insert	CGGGTTCCTTGGAAAAATCC
prEM0061	To amplify *wbpL* deletion insert	GGCACCCCAGGCTTTACACTTTATGCGACCGCCTTTGATCTATGC
prEM0062	To amplify *wbpL* deletion insert	GCTTAGGATTTTTCCAAGGAACCCGCGCGATCATCCAGATCATCAT
prEM0066	Verification of *wbpL* deletion	CTTGTAGTTGATCTGCACACC
prEM0067	Verification of *wbpL* deletion	CTATGCCATCTCCAAATACG
prEM0306	To amplify PA5001 deletion insert	TTGCGTTTTCCCTTGTCCAGATAGCTTCGATATAGACATCTTCCAGGC
prEM0307	To amplify PA5001 deletion insert	CTGATGAAAGTTTTTACCTGAGAGTAGCAGCCG
prEM0308	To amplify PA5001 deletion insert	CTCTCAGGTAAAAACTTTCATCAGACCTTCATCCTC
prEM0309	To amplify PA5001 deletion insert	GGCACCCCAGGCTTTACACTTTATGAACTGGGTTATTTCTGCCTGC
prEM0310	Verification of PA5001 deletion	TTCTTGTACGTATTGGTGGGAT
prEM0311	Verification of PA5001 deletion	GAGATCGAAACCGAGCATTAC
prEM0305	To amplify PA5001 region for complementatio n	GGCGTTACCCAACTTAATCGCCTTGAAGAATGCTCGCCGTTGCT

prEM0306	To amplify PA5001 region for complementation	ATCCGCCAAAACAGGGAATTTATGCCTTTTCGGCTGCTACTCTCAGGT
prEM0318	To amplify *galU* region for complementation	GGCGTTACCCAACTTAATCGCCTTGGTGACCGAGTGGAAACAGTTCC
prEM0319	To amplify *galU* region for complementation	ATCCGCCAAAACAGGGAATTTATGCGTTCGCCCCATACGAAAAACG
prAH2376	To linearise SC101-miniTn7-Gm-P*lac-QI*	GCATAAATTCCCTGTTTTGG
prAH2319	To linearise SC101-miniTn7-Gm-P*lac-QI*	CAAGGCGATTAAGTTGGGTAA
prAH2381	To amplify pUC18-miniTn7-Gm for *ori* exchange with SC101	CACGTTAAGGGATTTTGGTC
prAH2382	To amplify pUC18-miniTn7-Gm for *ori* exchange with SC101	GGGTCATTATAGCGATTTTTC
prAH2379	To amplify SC101 *ori* to insert in linearized pUC18-miniTn7-Gm	ACCGAAAAAATCGCTATAATGACCCCTCCTGTTGATAGATCCAGTAATG
prAH2380	To amplify SC101 *ori* to insert in linearized pUC18-miniTn7-Gm	CTCATGACCAAAATCCCTTAACGTGCCGCTGTAACAAGTTGTCTC
prAH2391:	To linearise SC101-miniTn7-Gm for insertion of P*lac-lacIQ*	GCACCCCAGGCTTTACACTTTATGCCTGAGTAGGACAAATCCGCC

prAH2371	To linearise SC101-miniTn7-Gm for insertion of P*lac-lacIQ*	ACAGGAAGCAAAGCTGAAAGGAATCCGTTTAAGGG CACCAATAACTG
prAH2201	To exchange ATG to GTG of *sce-I* gene of pFOG	AGAGAAAAGTGAAGTGCATCAAAAAAACCAGGTA
prAH2374	To amplify P*lac-lacIQ* from pAH186SC101_e	GATTCCTTTCAGCTTTGCTTC
prAH2375	To amplify P*lac-lacIQ* from pAH186SC101_e	GCATAAAGTGTAAAGCCTGGG
prAH2202	To exchange ATG to GTG of *sce-I* gene of pFOG	TGCACTTCACTTTTCTCTATCACTGATAGG
prEM0336	To amplify the NDM beta-lactamase and its promoter region	GAATCCAAGCTAGACTGCGATGGACCCACATTCACC CTGGC
prEM0337	To amplify the NDM beta-lactamase and its promoter region	CGTTTAAGGGCACCAATAACTGTCAGCGCAGCTTGT CGG
prEM0345	To amplify homology regions around *oprD*	TTGCGTTTTCCCTTGTCCAGATAGCAAAGTCGCCGA GCAACAGG
prEM0346	To amplify homology regions around *oprD*	CATCACTTTCATTGTGATTGCTCC
prEM0347	To amplify the *oprD* allele of EM-460	GAGCAATCACAATGAAAGTGATG
prEM0348	To amplify the *oprD* allele of EM-460	CGGTCGATTACAGGATCGACAGCG
prEM0349	To amplify homology regions around *oprD*	CGCTGTCGATCCTGTAATCGACCG

prEM0350	To amplify homology regions around *oprD*	GGCACCCCAGGCTTTACACTTTATGCATTTCCTCGCGGACGATG
prEM0324	To screen for *oprD_CI* insertion	AAGAAGAACTAGCCGTCACTGC
prEM0325	To screen for *oprD_CI* insertion	GTTTTTTCGTTGCCTGTCG
prEM0295	To amplify upstream homology for deleting PA1716 (*pscC*)	TTGCGTTTTCCCTTGTCCAGATAGCGAATGTCTTCCATGCTCCGT
prEM0296	To amplify upstream homology for deleting PA1716 (*pscC*)	CGCCGCCTGCTGAATTAGCATGGCCTGGAAGA
prEM0297	To amplify upstream homology for deleting PA1716 (*pscC*)	GCCATGCTAATTCAGCAGGCGGCGCATCAGG
prEM0298	To amplify upstream homology for deleting PA1716 (*pscC*)	GGCACCCCAGGCTTTACACTTTATGCAGGCGCGCACCCAGGAACA
prEM0299	To verify deletion of PA1716 (*pscC*)	GATAGCCGAATTTCTGCAGG
prEM0300	To verify deletion of PA1716 (*pscC*)	ACTGCGAGTCAAGTCCGAAT
prEM0177	To linearize SC101miniTn7 Gm	CATCGCAGTCTAGCTTGGATTC
prEM0178	To linearize SC101miniTn7 Gm	CAGTTATTGGTGCCCTTAAACG

Table S5 - Plasmids used in this study

Plasmid	Description	Reference
pEX18-Tc	suicide vector for allelic exchange in *P. aeruginosa*	Hoang et al., *Gene* (1998)[20]
pEX18-Tc_*hsdR17*	suicide vector used to generate *hsdR17* mutation to inactivate *hsdR* of PAO1	this study
pFOG	suicide vector for allelic exchange in *P. aeruginosa*	Cianfanelli et al., *BMC Microbiol* (2020)[11]
pFOGG	variant of pFOG in which the ATG start codon of I-SceI has been replaced with GTG	this study
pFOGG_*ΔrelA*	suicide vector used to generate *relA* deletion in PAO1	this study
pFOGG_*ΔspoT*	suicide vector used to generate *spoT* deletion in PAO1	this study
pFOGG_*ΔrpoS*	suicide vector used to generate *rpoS* deletion in PAO1	this study
pFOGG_*ΔgalU*	suicide vector used to generate *galU* deletion in PAO1	this study
pFOGG_*ΔwbpL*	suicide vector used to generate *wbpL* deletion in PAO1	this study
pFOGG_PA5001/*ssg*	suicide vector used to generate *ssg* deletion in PAO1	this study
pFOGG_*oprD_CI*	suicide vector used to introduce the *oprD_CI* allele of EM-490 into PAO1	this study
pFOGG_ *ΔpscC*	suicide vector used to generate *pscC* deletion in PAO1	this study
SC101miniTn7-Gm-Plac_*galU*	complementation of *galU* mutants	this study
SC101miniTn7-Gm-Plac_PA5001	complementation of PA5001 mutant	this study
SC101miniTn7-Gm_NDM	To introduce an NDM beta-lactamase and the 300bp upstream from the template strain (EM-460) into PAO1.	this study
pTNS2	Helper plasmid for transformation of miniTn7 based plasmids	Choi et al., *Nat Methods* (2006)[12]
pFLP2	FRT/FLP excising plasmid for removal of antibiotic cassette of miniTn7 based constructs	Hoang et al., *Gene* (1998)[10]
pUC18miniTn7_ev	Empty plasmid used as control	Choi et al., *Nat Methods* (2005)[12]
miniCTX_ev	Empty plasmid used as control	Hoang et al., *Plasmid* (2000)[13]

Table S6 – Minimum inhibitory concentrations of antibiotics for clinical isolates

Table S6. ***MIC values of tobramycin and ciprofloxacin for P. aeruginosa PAO1 Δpel Δpsl, CI249 and CI282 in M9Rich medium****. The values reported are the average of two independent experiments.*

Strain	Tobramycin sulfate MIC [µg/mL]	Ciprofloxacin MIC [µg/mL]
PAO1 *ΔpelΔpsl*	1	0.125
CI249	2	0.25
CI282	>32	>4

Table S7 – Minimum inhibitory concentration of meropenem for a resistant strain

Table S7. ***MIC values of P. aeruginosa PAO1 Δpel Δpsl and P. aeruginosa PAO1 Δpel Δpsl oprD_CI NDM*** *in M9Glucose medium. The values are the average of two independent experiments.*

Strain	Meropenem trihydrate MIC [µg/mL]
PAO1 *ΔpelΔpsl*	0.5
PAO1 *ΔpelΔpsl oprD_CI NDM*	8

Supplementary Note 1 – interpretation of free virion PFU increase for phages infecting dormant *Pseudomonas aeruginosa* cultures

An interesting phenomenon observed with deep-dormant cultures is that some *Pseudomonas aeruginosa* phages seem to rapidly adsorb and enter hibernation after which the number of free virions increases again over the next days, but never above the inoculum (Fig. 1d, Fig. 2b). In the absence of evidence for viral replication or bacterial killing it seems evident that this phenomenon is caused by reversible adsorption of a subpopulation of phages that fail to irreversibly bind the terminal surface receptor and inject their genome. This would be reminiscent of *E. coli* phages T1 or phi80 that can only irreversibly bind their FhuA receptor and inject their genome when FhuA is energized by the TonB system[14]. However, the different phages showing this behaviour in our study bind to different receptors on the cell surface with phiKZ and DMS3*vir* targeting type IV pili while PB1-like phages like UT1 generally target the *P. aeruginosa* LPS[4,15]. Thus, this phenomenon could instead be caused by phenotypic resistance of a bacterial subpopulation to irreversible phage adsorption or by phenotypic heterogeneity on the phage side as observed previously in other contexts[16].

Supplementary References

1 Maffei, E., Fino, C. & Harms, A. Antibiotic Tolerance and Persistence Studied Throughout Bacterial Growth Phases. *Methods Mol Biol* **2357**, 23-40, doi:10.1007/978-1-0716-1621-5_2 (2021).

2 Meadow, P. M. & Wells, P. L. Receptor Sites for R-type Pyocins and Bacteriophage E79 in the Core Part of the Lipopolysaccharide of *Pseudomonas aeruginosa* PAC1. *Microbiology* **108**, 339-343, doi:https://doi.org/10.1099/00221287-108-2-339 (1978).

3 Budzik, J. M., Rosche, W. A., Rietsch, A. & O'Toole, G. A. Isolation and characterization of a generalized transducing phage for *Pseudomonas aeruginosa* strains PAO1 and PA14. *J Bacteriol* **186**, 3270-3273, doi:10.1128/JB.186.10.3270-3273.2004 (2004).

4 Markwitz, P. *et al.* Genome-driven elucidation of phage-host interplay and impact of phage resistance evolution on bacterial fitness. *ISME J* **16**, 533-542, doi:10.1038/s41396-021-01096-5 (2022).

5 Maffei, E. *et al.* Systematic exploration of *Escherichia coli* phage-host interactions with the BASEL phage collection. *PLoS Biol* **19**, e3001424, doi:10.1371/journal.pbio.3001424 (2021).

6 Schrader, H. S. *et al.* Bacteriophage infection and multiplication occur in *Pseudomonas aeruginosa* starved for 5 years. *Can J Microbiol* **43**, 1157-1163 (1997).

7 Krylov, V. N. & Zhazykov, I. [Pseudomonas bacteriophage phiKZ--possible model for studying the genetic control of morphogenesis]. *Genetika* **14**, 678-685 (1978).

8 Broder, U. N., Jaeger, T. & Jenal, U. LadS is a calcium-responsive kinase that induces acute-to-chronic virulence switch in *Pseudomonas aeruginosa*. *Nat Microbiol* **2**, 16184, doi:10.1038/nmicrobiol.2016.184 (2016).

9 Laventie, B. J. *et al.* A Surface-Induced Asymmetric Program Promotes Tissue Colonization by *Pseudomonas aeruginosa*. *Cell Host Microbe* **25**, 140-152 e146, doi:10.1016/j.chom.2018.11.008 (2019).

10 Hoang, T. T., Karkhoff-Schweizer, R. R., Kutchma, A. J. & Schweizer, H. P. A broad-host-range Flp-FRT recombination system for site-specific excision of chromosomally-located DNA sequences: application for isolation of unmarked *Pseudomonas aeruginosa* mutants. *Gene* **212**, 77-86, doi:10.1016/s0378-1119(98)00130-9 (1998).

11 Cianfanelli, F. R., Cunrath, O. & Bumann, D. Efficient dual-negative selection for bacterial genome editing. *BMC Microbiol* **20**, 129, doi:10.1186/s12866-020-01819-2 (2020).

12 Choi, K. H. & Schweizer, H. P. mini-Tn7 insertion in bacteria with single attTn7 sites: example *Pseudomonas aeruginosa*. *Nat Protoc* **1**, 153-161, doi:10.1038/nprot.2006.24 (2006).

13 Hoang, T. T., Kutchma, A. J., Becher, A. & Schweizer, H. P. Integration-proficient plasmids for *Pseudomonas aeruginosa*: site-specific integration and use for engineering of reporter and expression strains. *Plasmid* **43**, 59-72, doi:10.1006/plas.1999.1441 (2000).

14 Braun, V. FhuA (TonA), the career of a protein. *J Bacteriol* **191**, 3431-3436, doi:10.1128/JB.00106-09 (2009).

15 Wright, R. C. T., Friman, V. P., Smith, M. C. M. & Brockhurst, M. A. Cross-resistance is modular in bacteria-phage interactions. *PLoS Biol* **16**, e2006057, doi:10.1371/journal.pbio.2006057 (2018).

16 Gallet, R., Lenormand, T. & Wang, I. N. Phenotypic stochasticity protects lytic bacteriophage populations from extinction during the bacterial stationary phase. *Evolution* **66**, 3485-3494, doi:10.1111/j.1558-5646.2012.01690.x (2012).
