# Supplementary material for: Phage Paride can kill dormant, antibiotic-tolerant cells of Pseudomonas aeruginosa by direct lytic replication
Source: Nat Commun. 2024 Jan 2;15:175. doi: 10.1038/s41467-023-44157-3 (PMC10761892; doi:10.1038/s41467-023-44157-3)
Supplement: Supplementary file 3 — Description of Additional Supplementary Files [file 41467_2023_44157_MOESM3_ESM.pdf]

Description of Additional Supplementary Files:

Supplementary Data 1: Construction of all plasmids generated in this study. Plasmids have been generated by restriction-ligation cloning or Gibson Assembly using templates and oligonucleotides as listed in this table. Additional information about procedures is available in the Methods section.

Supplementary Data 2: Clinical *Pseudomonas aeruginosa* strains used in this study. This table lists clinical isolates of *P. aeruginosa* that were tested for Paride sensitivity and their drug resistances (if known). Based on these results, the strains used in Fig. 3A,B have been selected.
