# Supplementary material for: Phage Paride can kill dormant, antibiotic-tolerant cells of Pseudomonas aeruginosa by direct lytic replication
Source: Nat Commun. 2024 Jan 2;15:175. doi: 10.1038/s41467-023-44157-3 (PMC10761892; doi:10.1038/s41467-023-44157-3)
Supplement: Supplementary file 6 — Reporting Summary [file 41467_2023_44157_MOESM6_ESM.pdf]

nature portfolio

Corresponding author(s): Prof. Dr. Alexander Harms

Last updated by author(s): Nov 27, 2023

Reporting Summary

Nature Portfolio wishes to improve the reproducibility of the work that we publish. This form provides structure for consistency and transparency in reporting. For further information on Nature Portfolio policies, see our Editorial Policies and the Editorial Policy Checklist.

Statistics

For all statistical analyses, confirm that the following items are present in the figure legend, table legend, main text, or Methods section.

n/a	Confirmed	
☐	☒	The exact sample size (n) for each experimental group/condition, given as a discrete number and unit of measurement
☐	☒	A statement on whether measurements were taken from distinct samples or whether the same sample was measured repeatedly
☐	☒	The statistical test(s) used AND whether they are one- or two-sided<br>*Only common tests should be described solely by name; describe more complex techniques in the Methods section.*
☐	☒	A description of all covariates tested
☐	☒	A description of any assumptions or corrections, such as tests of normality and adjustment for multiple comparisons
☒	☐	A full description of the statistical parameters including central tendency (e.g. means) or other basic estimates (e.g. regression coefficient) AND variation (e.g. standard deviation) or associated estimates of uncertainty (e.g. confidence intervals)
☒	☐	For null hypothesis testing, the test statistic (e.g. F, t, r) with confidence intervals, effect sizes, degrees of freedom and P value noted<br>*Give P values as exact values whenever suitable.*
☒	☐	For Bayesian analysis, information on the choice of priors and Markov chain Monte Carlo settings
☒	☐	For hierarchical and complex designs, identification of the appropriate level for tests and full reporting of outcomes
☒	☐	Estimates of effect sizes (e.g. Cohen's d, Pearson's r), indicating how they were calculated

Our web collection on statistics for biologists contains articles on many of the points above.

Software and code

Policy information about availability of computer code

Data collection	No software was used to collect the quantitative data reported in this study.
Data analysis	Data were analysed in Microsoft Excel and plotted using R-Studio. The R-studio code used for the analysis of CFU and PFU curve data is available at the Zenodo repository with the digital object identifier (DOI) 10.5281/zenodo.10063932 [https://doi.org/10.5281/zenodo.10063933].

For manuscripts utilizing custom algorithms or software that are central to the research but not yet described in published literature, software must be made available to editors and reviewers. We strongly encourage code deposition in a community repository (e.g. GitHub). See the Nature Portfolio guidelines for submitting code & software for further information.

Data

Policy information about availability of data

All manuscripts must include a data availability statement. This statement should provide the following information, where applicable:

- Accession codes, unique identifiers, or web links for publicly available datasets
- A description of any restrictions on data availability
- For clinical datasets or third party data, please ensure that the statement adheres to our policy

Most data generated or analysed during this study are included in this published article (including supplementary information and source data file). Genome sequences of all newly isolated and sequenced phages have been deposited in the NCBI GenBank repository. The genome of Pseudomonas phage Aergia has been deposited with accession OR805291, the genome of Pseudomonas phage Cassandra has been deposited with accession OR805292, the genome of Pseudomonas

phage Deifobo has been deposited with accession OR805293, the genome of Pseudomonas phage Ettore has been deposited with accession OR805294, the genome of Pseudomonas phage Paride has been deposited with accession OR805295, and the genome of Pseudomonas phage Victoria has been deposited with accession OR805296. Raw data of all proteomics experiments have been deposited in the ProteomeXchange database and the MassIVE repository under accession codes PXD041131 and MSV000091557, respectively.

Research involving human participants, their data, or biological material

Policy information about studies with human participants or human data. See also policy information about sex, gender (identity/presentation), and sexual orientation and race, ethnicity and racism.

Reporting on sex and gender	No human research participants were used in this study.
Reporting on race, ethnicity, or other socially relevant groupings	No human research participants were used in this study.
Population characteristics	No human research participants were used in this study.
Recruitment	No human research participants were used in this study.
Ethics oversight	No human research participants were used in this study.

Note that full information on the approval of the study protocol must also be provided in the manuscript.

Field-specific reporting

Please select the one below that is the best fit for your research. If you are not sure, read the appropriate sections before making your selection.

[x] Life sciences [ ] Behavioural & social sciences [ ] Ecological, evolutionary & environmental sciences

For a reference copy of the document with all sections, see nature.com/documents/nr-reporting-summary-flat.pdf

Life sciences study design

All studies must disclose on these points even when the disclosure is negative.

Sample size	Sample sizes were chosen to be the minimum for meaningful statistical analyses (i.e., n=3 or higher) which provided robust differences or similarities between conditions and sample groups.
Data exclusions	No data were excluded.
Replication	At least three independent biological replicates were performed per experiment. All replicates were successful and are shown (with the exception of, e.g., uninterpretable data in case of broken culture tubes or mice with failed bacterial colonization).
Randomization	Tubes of bacterial culturing experiments were randomized in treatment order. Mice were randomly allocated to treatment groups for the tissue cage infection experiments.
Blinding	Blinding of the experimentor was not possible during direct experimentation because samples were directly pipetted.

Reporting for specific materials, systems and methods

We require information from authors about some types of materials, experimental systems and methods used in many studies. Here, indicate whether each material, system or method listed is relevant to your study. If you are not sure if a list item applies to your research, read the appropriate section before selecting a response.

Materials & experimental systems

n/a	Involved in the study	
x		Antibodies
x		Eukaryotic cell lines
x		Palaeontology and archaeology
	x	Animals and other organisms
x		Clinical data
x		Dual use research of concern
x		Plants

Methods

n/a	Involved in the study	
x		ChIP-seq
x		Flow cytometry
x		MRI-based neuroimaging

Animals and other research organisms

Policy information about studies involving animals; ARRIVE guidelines recommended for reporting animal research, and Sex and Gender in Research

Laboratory animals	13-week old female C57BL/6 mice (minimum weight 20g) were used for our experiments (more details on housing etc. are provided in the dedicated methods section under "Tissue cage infection experiments").
Wild animals	No wild animals were used.
Reporting on sex	All mice were female due to the higher aggressiveness of male mice who tend to scratch open the implanted tissue cages more often than the females (for this reason also the animal permit for these experiments only covers female animals).
Field-collected samples	No relevant field samples were used.
Ethics oversight	Animal experiments were performed according to the regulations of Swiss veterinary law (permit number #1710) and the licence has been approved by the cantonal veterinary office of Basel-Stadt (Switzerland).

Note that full information on the approval of the study protocol must also be provided in the manuscript.

Plants

Seed stocks	No plants were used in this study.
Novel plant genotypes	No plants were used in this study.
Authentication	No plants were used in this study.
